# Matrix nanoalignment programs macrophage vesicular immunity through TRPV2 mechanogating

**DOI:** 10.1016/j.bioactmat.2026.09.011

**Published:** 2026-09-23

**Authors:** Bohan Yin, Guangli Xiang, Ziqi Zhang, Limeng Zheng, Runyao Wang, Qin Zhang, Yingying Huang, Chenjie Liu, Jingjing Chi, Xinyue Xia, Jiaxiang Yan, Jianfang Wang, Mo Yang, Liming Bian, Siu Hong Dexter Wong

**Affiliations:** a School of Medicine and Pharmacy, Ocean University of China, Qingdao, 266100, China; b Laboratory for Marine Drugs and Bioproducts, Qingdao Marine Science and Technology Center, Qingdao, 266237, China; c Department of Biomedical Engineering, The Hong Kong Polytechnic University, Hong Kong, 999077, China; d School of Biomedical Sciences and Engineering, South China University of Technology, Guangzhou International Campus, Guangzhou, 511442, China; e Department of Physics, The Chinese University of Hong Kong, Hong Kong, 999077, China

**Keywords:** ECM nanoarchitecture, Tumor-associated macrophages, Mechanobiology, Small extracellular vesicle release, Magnetic control

## Abstract

The extracellular matrix (ECM) undergoes extensive architectural remodeling during tumor progression, generating nanoscale alignment cues that profoundly influence behaviors of immune cells. However, whether ECM nanoarchitecture directly programs their immune secretory functions remains poorly defined. Here, we develop a magnetically switchable hydrogel that enables reversible, on-demand modulation of nanoscale ligand alignment without altering bulk stiffness or composition. Using this platform, we uncover a mechanotransduction pathway in tumor-associated macrophages (TAMs) whereby matrix nanoalignment enhances integrin β1-dependent mechanosensing and transient receptor potential cation channel subfamily V member 2 (TRPV2)-dependent Ca^2+^ signaling, thereby promoting phosphoinositide 3-kinase (PI3K)–Ras-related protein 11a (Rab11a)-associated small extracellular vesicle (sEV) release by approximately tenfold. Beyond increasing vesicle output, PI3K–mechanistic target of rapamycin complex 2-associated signaling altered the particle-normalized cargo profile of sEVs, with preferential enrichment of immunoregulatory molecules, including M2-promoting cytokines and miR-181a-5p. These sEVs reprogram macrophages toward an immunosuppressive M2-like phenotype, suppress cytotoxic CD8^+^ T-cell infiltration, and accelerate melanoma progression in vivo. Importantly, selective disruption of the TRPV2–Rab11a axis or pharmacological attenuation of ECM remodeling restores antitumor immunity and restricts tumor growth. Together, our findings demonstrate that ECM nanoarchitecture is sufficient to regulate macrophage sEV programming in a defined matrix environment and identify TRPV2-associated mechanotransduction as a potential therapeutic vulnerability in matrix-remodeled tumors.

## Highlights

• Nanoaligned ECM programs TAM immunosuppressive communication.
• TRPV2 links ECM nanoarchitecture to Rab11a-dependent sEV release.
• Rab11a and mTORC2 regulate sEV abundance and immunoregulatory cargo.
• NAM-primed sEVs suppress TLR4–NF-κB inflammatory signaling.
• TAM-targeted mechanosensitive sEV programming restores antitumor immunity in melanoma.

## Graphical abstract

**Figure:**
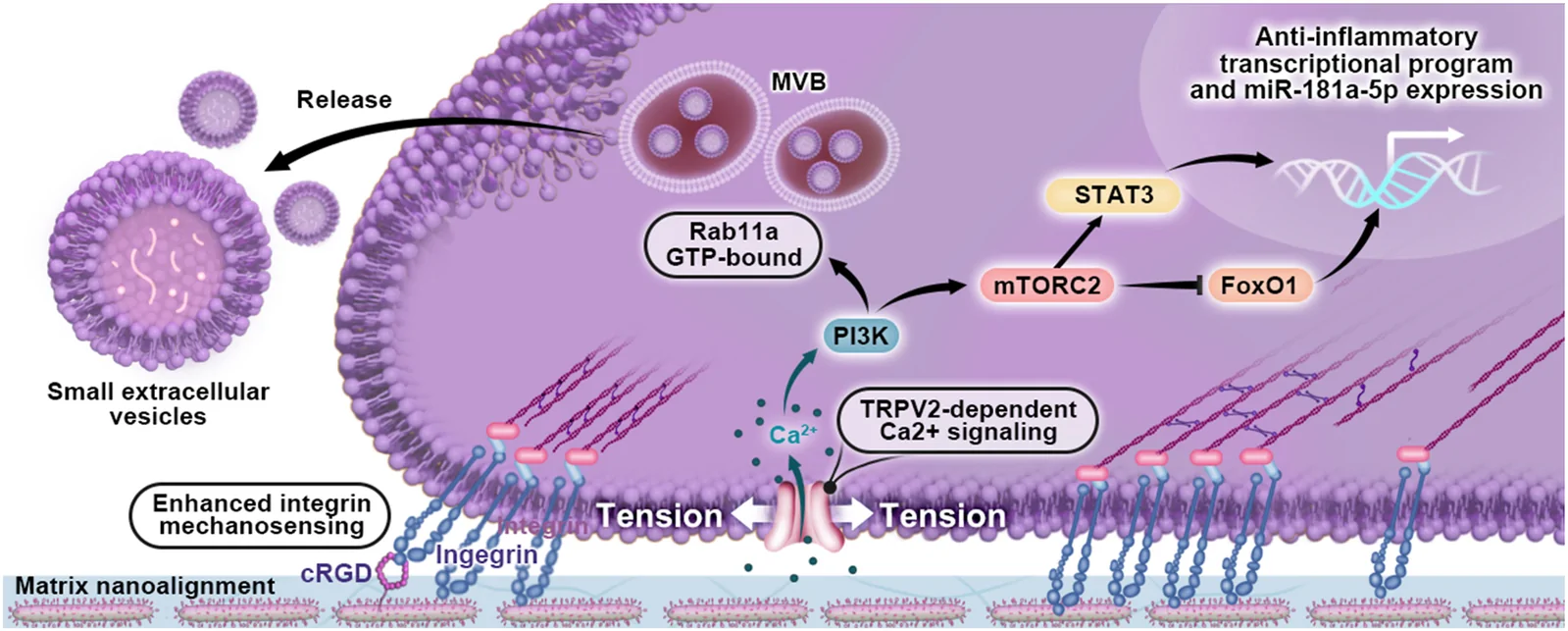

## 1 Introduction

The tumor microenvironment (TME) is shaped not only by its cellular heterogeneity but also by the dynamic and evolving architecture of the extracellular matrix (ECM), which undergoes extensive remodeling during tumor progression [[1], [2], [3]]. Progressive desmoplasia alters matrix density, fiber alignment, and topographical anisotropy, generating complex biophysical cues that regulate cancer cell behavior, immune infiltration, and therapeutic response [[4], [5], [6], [7], [8]]. Although the effects of ECM stiffness, viscoelasticity, and porosity on cancer biology have been intensively studied [[9], [10], [11]], less is known about how nanoscale architectural features, such as ligand orientation and fibrillar alignment, instruct immune-cell function [12,13]. These structural cues reorganize adhesion geometry and force transmission at the cell–matrix interface, thereby activating signaling programs that are distinct from those induced by bulk matrix mechanics [[14], [15], [16]].

ECM alignment is increasingly recognized as a physical hallmark of aggressive tumors [17]. Collagen fiber anisotropy correlates with invasion, metastasis, immune exclusion, and poor therapeutic response across multiple cancer types. In melanoma, dense and aligned ECM is a prominent feature of advanced disease and has been linked to poor responsiveness to immune checkpoint blockade, increased macrophage infiltration, and immune suppression [18]. These observations suggest that ECM architecture, beyond matrix stiffness alone, may actively contribute to immune suppression in melanoma. However, because fibrotic tumor stroma contains intertwined biochemical, cellular, and mechanical changes, it remains difficult to determine whether nanoscale ECM alignment itself is sufficient to regulate behaviors of immune cells.

Tumor-associated macrophages (TAMs) are among the most abundant stromal immune cells in melanoma and other solid tumors, where they regulate angiogenesis, matrix remodeling, immune suppression, and therapeutic resistance [19,20]. In collagen-rich tumor regions, TAMs often reside along fibrillar ECM tracks and adopt elongated morphologies characteristic of mechanically polarized cells [[21], [22], [23], [24]]. Sustained interactions with ECM and tumor-derived cues can drive TAMs toward immunosuppressive states that suppress cytotoxic T-cell activity and support tumor progression. One important route by which TAMs communicate with surrounding immune and stromal cells is through the release of small extracellular vesicles (sEVs), which carry proteins, cytokines, and regulatory microRNAs (miRNAs) [[25], [26], [27], [28], [29], [30], [31]]. TAM-derived sEVs can reinforce M2-like polarization, remodel inflammatory signaling, and promote tumor progression [32,33]. Although the molecular machinery of sEV biogenesis has been extensively studied, including endosomal sorting complexes required for transport (ESCRT)-dependent and ESCRT-independent pathways and Ras association domain-containing protein (Rab) GTPase-mediated vesicle trafficking [[34], [35], [36], [37]], whether nanoscale ECM alignment activates this machinery in TAMs through a defined mechanosensitive pathway remains unclear.

Mechanical perturbations such as shear stress, compression, and substrate rigidity can modulate EV release in several cell types [[38], [39], [40], [41]]. In particular, stiff ECM has been shown to stimulate tumor-cell EV secretion through an Akt–Rab8 pathway that promotes tumor growth [40]. However, these studies primarily address bulk mechanical inputs rather than nanoscale ligand alignment decoupled from matrix stiffness. This distinction is important because TAMs in fibrotic melanoma stroma encounter polarized adhesion, directional force transmission, and confined interstitial tracks generated by aligned ECM bundles [42,43]. Integrin-based adhesions are sensitive to such architectural features, and integrin trafficking is known to involve Rab-regulated recycling pathways [20,44]. Nevertheless, prior work has not clarified whether ECM nanoalignment gates Rab11a-dependent sEV release through mechanosensitive ion-channel activation in macrophages.

Here, using melanoma as a disease model, we test the hypothesis that nanoscale ECM alignment is sufficient to program TAM-derived sEV release and immunoregulatory function (Fig. 1a). To isolate this physical variable, we engineer a magnetically switchable hydrogel that enables reversible control of nanoscale ligand alignment while maintaining constant bulk stiffness and matrix composition (Fig. 1c). This reductionist platform intentionally omits the ligand heterogeneity, matrix-bound cytokines, degradability, and dynamic remodeling of native melanoma ECM. It is designed to test whether nanoscale adhesive-ligand alignment is sufficient to regulate TAM behavior within a defined cyclo-Arg-Gly-Asp (cRGD)-presenting environment, rather than to establish that nanoalignment produces an identical or quantitatively universal response across all native ECM contexts. We show that nanoscale ligand alignment enhances integrin β1-dependent mechanosensing and activates a transient receptor potential cation channel subfamily V member 2 (TRPV2)-dependent Ca^2+^ influx pathway that engages phosphoinositide 3-kinase (PI3K) signaling. Downstream of this axis, PI3K signaling promotes Rab11a activation to enhance TAM-derived sEV release and is associated with mTORC2 activation and altered STAT3/FoxO1 signaling, together with particle-normalized enrichment of immunoregulatory sEV cargo, including M2-associated cytokines and miR-181a-5p. The matrix nanoalignment-primed TAM-derived sEVs suppress Toll-like receptor 4 (TLR4)/NF-κB inflammatory signaling, skew macrophages toward an immunosuppressive phenotype, reduce cytotoxic CD8^+^ T-cell infiltration, and accelerate melanoma progression in vivo. Conversely, targeting the TRPV2–Rab11a axis or attenuating transforming growth factor beta (TGF-β)-associated matrix remodeling in M2-like TAMs reduces sEV release, restores antitumor immune features, and restricts melanoma growth. Together, these findings identify ECM nanoarchitecture as an active regulator of macrophage immune communication and reveal a TRPV2–PI3K-dependent bifurcated signaling program, in which Rab11a controls sEV release while mTORC2-associated signaling contributes to sEV cargo remodeling.

**Fig. 1:**
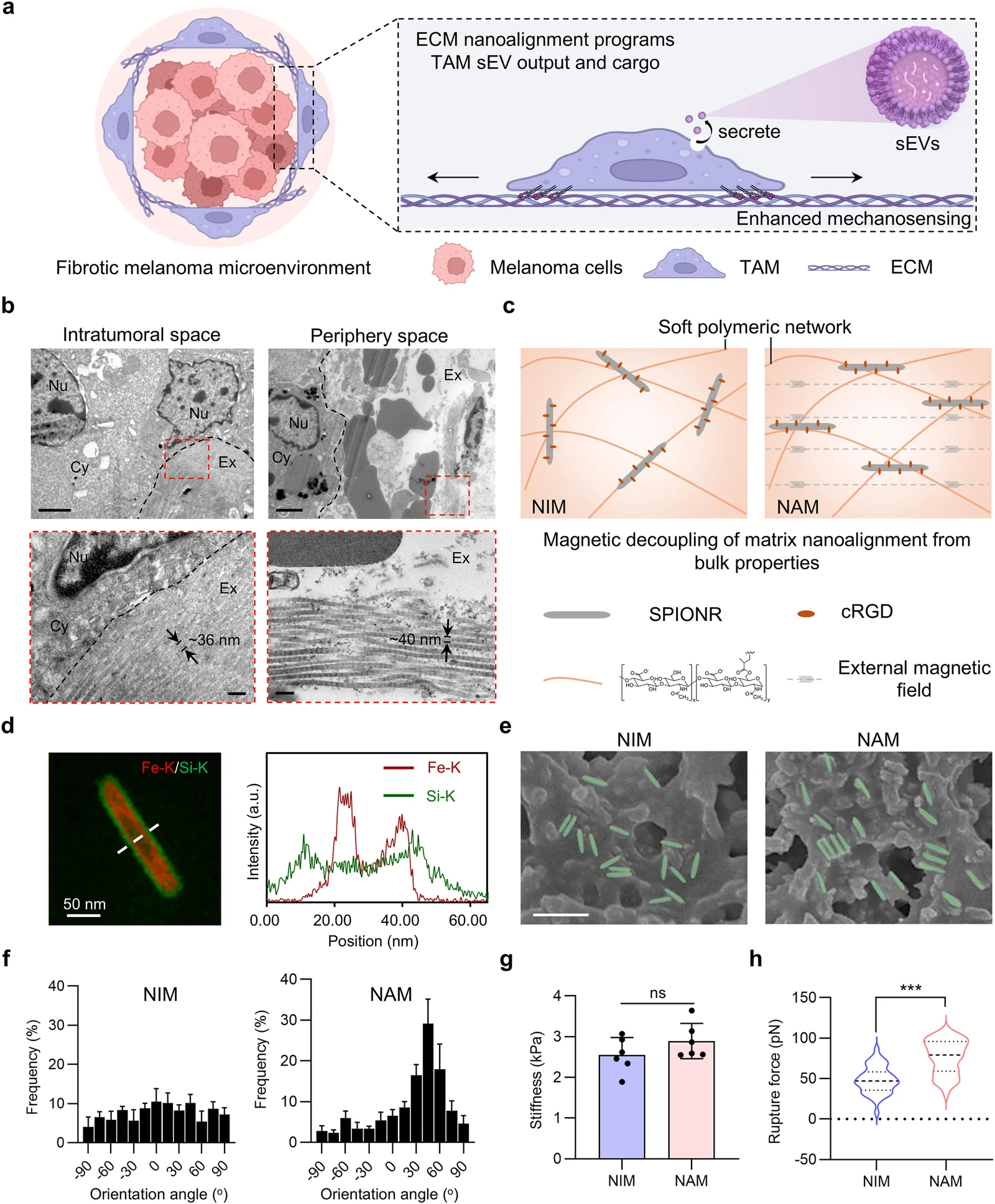
**Characterization of the aligned ECM niche in melanoma and a magnetically switchable matrix nanoalignment system for regulating macrophage mechanosensing and sEV biogenesis.** (a) Schematic illustration of aligned ECM architectures in melanoma that may enhance mechanosensing in attached tumor-associated macrophages (TAMs) and program their small extracellular vesicle (sEV) biogenesis and cargo. Parts of the figure were generated using BioRender.com. (b) TEM images showing aligned ECM structures in the intratumoral and peripheral regions of murine melanoma. Scale bars: 2 μm (left) and 200 nm (magnified view). (c) Schematic design of a magnetically switchable matrix that decouples nanoscale alignment from bulk matrix stiffness. The initial nonmagnetic state is a nanoscale isotropic matrix (NIM), whereas application of a lateral magnetic field induces a nanoscale aligned matrix (NAM). (d) HAADF-STEM images of nanofiber-mimetic nanorods (NMNs) with corresponding elemental mapping of Fe and Si (dashed outline). (e) SEM images of NIM and NAM. Scale bar: 200 nm. (f) Angular distributions of NMNs within NIM and NAM (n = 100 nanorods from three independent gels). (g) Stiffness measurements of NIM and NAM (n = 6). (h) Violin plots of average rupture forces measured on NIM and NAM (n = 40 from three independent gels). Data are presented as mean ± SD. Statistical significance was determined by unpaired *t*-test. ***p ≤ 0.001; ns, not significant.

## 2 Results & discussion

### 2.1 Fabrication and characterization of a magnetically switchable nanoaligned matrix

To investigate how nanoscale ECM architecture regulates macrophage behavior within tumors, we first characterized the ultrastructural features of the ECM in a murine B16-F10 syngeneic melanoma model. Transmission electron microscopy (TEM) revealed abundant, highly aligned fibrillar structures distributed throughout both intratumoral and peri-tumoral regions (Fig. 1b). These fibers exhibited diameters of approximately 36–40 nm, consistent with the nanoscale dimensions of fibrillar ECM components reported in solid tumors [45]. The prevalence of these aligned nanoscale fibers suggests that tumor ECM organization at length scales comparable to integrin nanoclusters may provide spatially encoded mechanical cues to infiltrating immune cells.

To model this architectural feature in a controllable and reversible manner, we synthesized nanofiber-mimetic nanorods (NMNs) that allow magnetic control of ligand nanoalignment independent of bulk matrix properties (Fig. S1a). TEM analysis revealed that the NMNs had an average length of 162 ± 22 nm and a width of 32 ± 6 nm, yielding an aspect ratio of approximately 5 (Fig. 1d and Fig. S1b). Notably, the nanorod width closely matched the diameter of the aligned fibrillar structures observed in melanoma ECM and overlapped with the reported size range of integrin nanoclusters [46], allowing direct interrogation of integrin-ligand interactions at physiologically relevant nanoscale dimensions.

Magnetic characterization using vibrating-sample magnetometry demonstrated that NMNs exhibited a high saturation magnetization (38.6 emu g^−1^) with negligible hysteresis, indicative of superparamagnetic behavior suitable for reversible alignment without remanent magnetization-induced aggregation (Fig. S1c). X-ray diffraction confirmed lattice parameters consistent with Fe_3_O_4_, further validating the magnetic core composition (Fig. S2). Stepwise changes in zeta potential during surface modification verified successful functionalization at each synthetic stage (Fig. S1d). High-angle annular dark-field scanning transmission electron microscopy (HAADF-STEM) combined with energy-dispersive X-ray spectroscopy mapping further confirmed a well-defined core–shell architecture, consisting of an iron-rich magnetic core encapsulated within a silica shell (Fig. 1d), which provides chemical stability and functionalization versatility.

NMNs were incorporated into a hyaluronic acid (HA)-based hydrogel matrix using thiol–ene click chemistry, following our established design strategy (Fig. S3) [2]. The surface density of NMNs was optimized to 95 ± 8 particles μm^−2^ to maintain sufficient ligand presentation while avoiding steric hindrance that could impair magnetic reorientation. Integrin-binding cRGD peptides were covalently conjugated to immobilized NMNs, as confirmed by a decrease in zeta potential, the emergence of characteristic amide bonds in Fourier-transform infrared spectra (Fig. S1d and S4), and a measured ligand density of 0.78 ± 0.12 peptides nm^−2^.

Recent stimuli-responsive nanomaterials and hydrogels have enabled spatiotemporal control of therapeutic activation and tissue responses [[47], [48], [49], [50]]. Scanning electron microscopy (SEM) revealed that NMNs within the hydrogel could be reoriented from a random, isotropic distribution, termed the nanoscale isotropic matrix (NIM), to a highly aligned configuration, termed the nanoscale aligned matrix (NAM), upon application of a lateral magnetic field within 1 h (Fig. 1e and f). Importantly, bulk mechanical testing demonstrated that magnetic alignment did not significantly alter the elastic modulus of the hydrogel (2.89 ± 0.43 kPa with magnetic field versus 2.48 ± 0.50 kPa without magnetic field (Fig. 1g), swelling ratio (Fig. S5a) and pore size (Fig. S5b), confirming that nanoscale ligand anisotropy was effectively decoupled from bulk physical properties. Atomic force microscopy-based single-molecule force spectroscopy confirmed that NAM increased integrin β1–ligand rupture forces relative to NIM (Fig. 1h), whereas overall integrin-binding ligand accessibility and local Young's modulus remained comparable between the two matrices (Fig. S6 and S7). These findings support enhanced integrin β1 engagement arising from matrix nanoalignment rather than altered ligand abundance or hydrogel stiffness. Together, these results establish a magnetically responsive ECM-mimicking matrix platform that models the ∼40 nm-scale aligned features observed in melanoma ECM while enabling selective control of nanoscale ligand organization as an upstream physical cue for TAM mechanotransduction under physiologically soft matrix conditions.

### 2.2 Matrix nanoalignment enhances adhesion and mechanosensing in TAMs

We next examined how tumor-associated macrophages (TAMs) respond to matrix nanoalignment. TAMs isolated from syngeneic melanoma tumors were cultured on NIM or NAM hydrogels, respectively (Fig. S8). Cell viability exceeded 90% under both conditions after 24 h, confirming cytocompatibility (Fig. 2a). Despite comparable viability, TAM adhesion was significantly enhanced on NAM, with 57.2 ± 5.9% of cells adhering within 24 h, approximately 1.5-fold higher than on NIM. Blocking integrin β1 reduced adhesion on NAM by more than 50%, whereas blockade of CD44 or isotype control antibodies exerted minimal effects (Fig. 2a and b), indicating that the enhanced macrophage adhesion on NAM is predominantly mediated by promoted integrin–ligand engagement rather than HA receptor engagement.

**Fig. 2:**
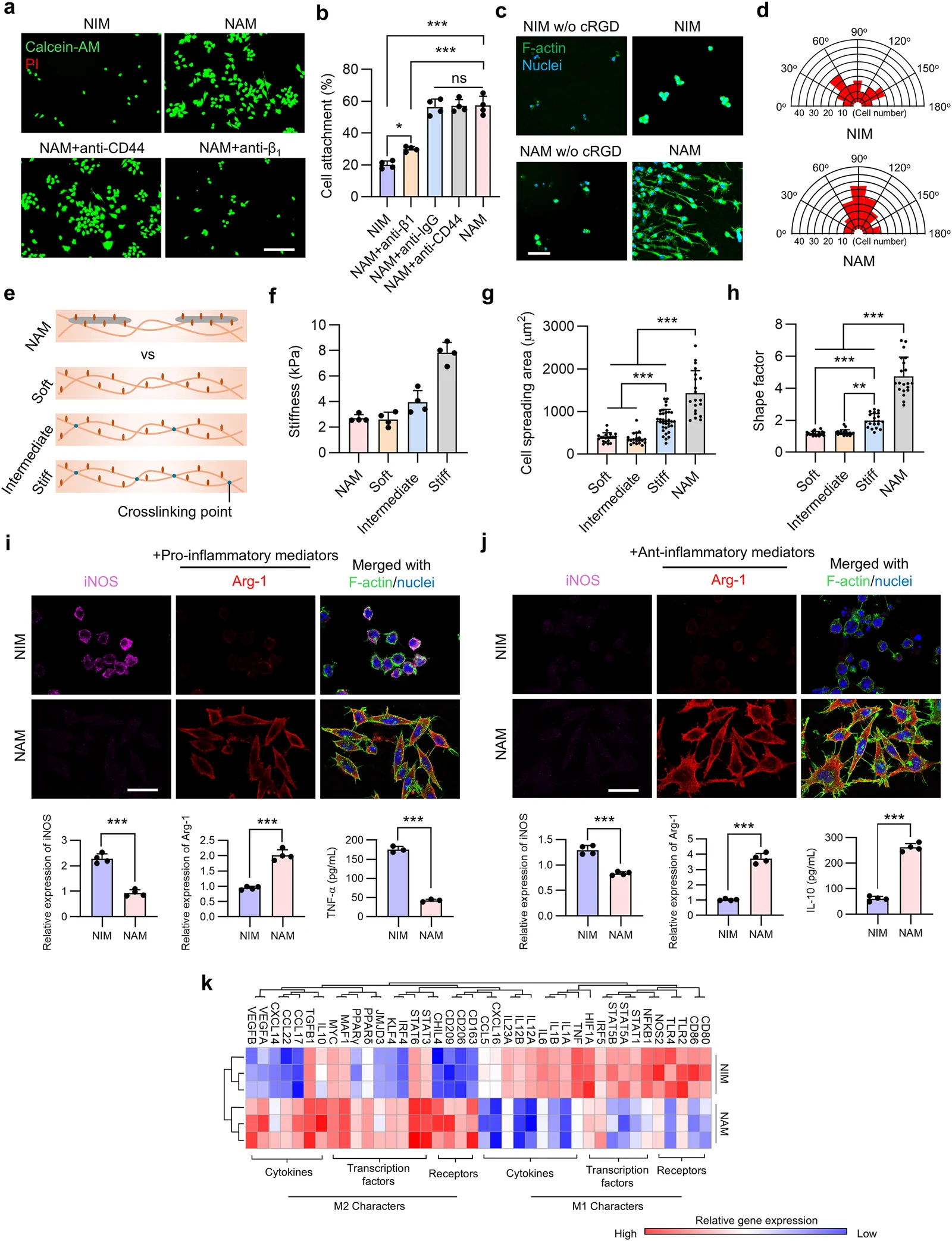
**Matrix nanoalignment enhances TAM mechanosensing and polarization more effectively than bulk matrix stiffness.** (a) Calcein-AM/propidium iodide (PI) staining of macrophages cultured on NIM or NAM for 24 h, with or without immunoblockade of CD44 (anti-CD44) or integrin β1 (anti-β1). Scale bar: 200 μm. (b) Quantification of macrophage attachment (n = 4). (c) F-actin stress fiber staining of macrophages cultured on NIM or NAM with or without (w/o) cRGD coupling. Scale bar: 50 μm. (d) Rose plots showing peak orientation angles of macrophages cultured on NIM or NAM; the magnetic field direction was set at 90°. (e) Schematic comparison of the regulatory effects of NAM and cRGD-coupled matrices with stiffness ranging from soft to stiff on macrophage adhesion. (f) Corresponding stiffness measurements (n = 4). (g) Quantification of cell spreading and (h) shape factor after 24 h of incubation (n = 20 cells from three independent hydrogels). Scale bar: 50 μm. (i,j) Confocal images of iNOS and Arg-1 immunostaining with corresponding gene expression (n = 4) and cytokine secretion analysis (TNF-α and IL-10, n = 4) under pro-inflammatory (i) or anti-inflammatory (j) conditions. (k) Hierarchical clustering heatmap of M1-and M2-associated gene expression (row-normalized Z-scores; n = 3). Data are presented as mean ± SD. Statistical significance was determined by one-way ANOVA with Tukey's multiple-comparison test. *p ≤ 0.05, **p ≤ 0.01, ***p ≤ 0.001; ns, not significant.

Matrix nanoalignment also promoted pronounced cytoskeletal polarization. TAMs cultured on NAM developed elongated morphologies with aligned F-actin stress fibers oriented along the magnetic field direction, whereas cells on NIM remained poorly spread and rounded (Fig. 2c,d,g,h and Fig. S9). In contrast, TAMs seeded on both NIM and NAM without cRGD conjugation showed minimal cell attachment (Fig. 2c and d). Notably, increasing the bulk stiffness of NIM hydrogels to 4 or 8 kPa failed to recapitulate the adhesive and cytoskeletal phenotypes observed on NAM (Fig. 2e–h), underscoring that nanoscale ligand alignment, rather than bulk rigidity, is a predominant determinant of macrophage mechanosensing in this defined system [51].

### 2.3 Matrix nanoalignment skews TAM polarization toward an M2-like phenotype

Given the established coupling between cytoskeletal tension and macrophage polarization [52], we examined whether nanoscale ligand alignment influences TAM functional states. Under pro-inflammatory conditions, TAMs cultured on NIM exhibited elevated inducible nitric oxide synthase (iNOS) expression and increased secretion of tumor necrosis factor-α (TNF-α), while maintaining low levels of arginase-1 (Arg-1) and interleukin-10 (IL-10) (Fig. 2i). In contrast, TAMs on NAM instead displayed significantly elevated Arg-1 expression, indicating a shift toward an M2-like phenotype despite pro-inflammatory cues.

Under anti-inflammatory conditions, NAM further amplified Arg-1 expression by approximately fourfold and IL-10 secretion by threefold relative to NIM (Fig. 2j). Notably, TAMs on NIM failed to robustly acquire M2-like characteristics even in the presence of anti-inflammatory cytokines, suggesting that an adhesion-permissive and mechanosensitive microenvironment supports efficient M2-like polarization. Heatmap analysis of representative macrophage polarization-associated genes, including surface receptors, transcription factors and cytokines, indicated that NAM-primed TAMs selectively upregulated the majority of M2-associated genes with suppressed M1-like associated transcripts (Fig. 2k). Subcutaneous implantation of NAM also enhanced TAM adhesion, spreading, and M2 polarization in vivo (Fig. S10–11). Dynamic switching experiments further revealed that macrophage polarization was reversible and remained responsive to changes in matrix nanoalignment (Fig. S12), demonstrating that TAM phenotypes can be actively maintained by mechanical cues rather than irreversibly programmed.

### 2.4 Matrix nanoalignment promotes TAM-derived sEV release through TRPV2/PI3K–Rab11a signaling

To investigate whether matrix nanoalignment regulates TAM secretory communication, we first characterized sEVs isolated from TAM-conditioned media. To assess the reproducibility of the isolation procedure, aliquots of differential ultracentrifugation-isolated sEVs were further purified by size-exclusion chromatography (SEC). Nanoparticle tracking analysis (NTA) showed a comparable particle-size distribution and no significant loss of particles within the sEV-size range after SEC purification (Fig. S13), indicating that the principal particle population was retained after orthogonal purification. Therefore, unless otherwise indicated, differential ultracentrifugation was used for sEV isolation in subsequent experiments. Compared with NIM-primed TAMs, sEVs derived from NAM-primed TAMs showed robust expression of EV-associated proteins, including CD63, CD81, and CD9 (Fig. 3a). Consistently, NTA indicated that NAM-primed TAMs released more than tenfold higher quantities of sEVs within the 30–150 nm size range than NIM-primed TAMs after normalization to the number of viable adherent donor cells at conditioned-medium collection (Fig. 3b and c). TEM further revealed abundant vesicle-like structures associated with the plasma membrane of NAM-cultured TAMs, whereas NIM-cultured TAMs exhibited sparse vesicular structures (Fig. 3d). These results indicate that nanoscale matrix alignment substantially enhances TAM-derived sEV release.

**Fig. 3:**
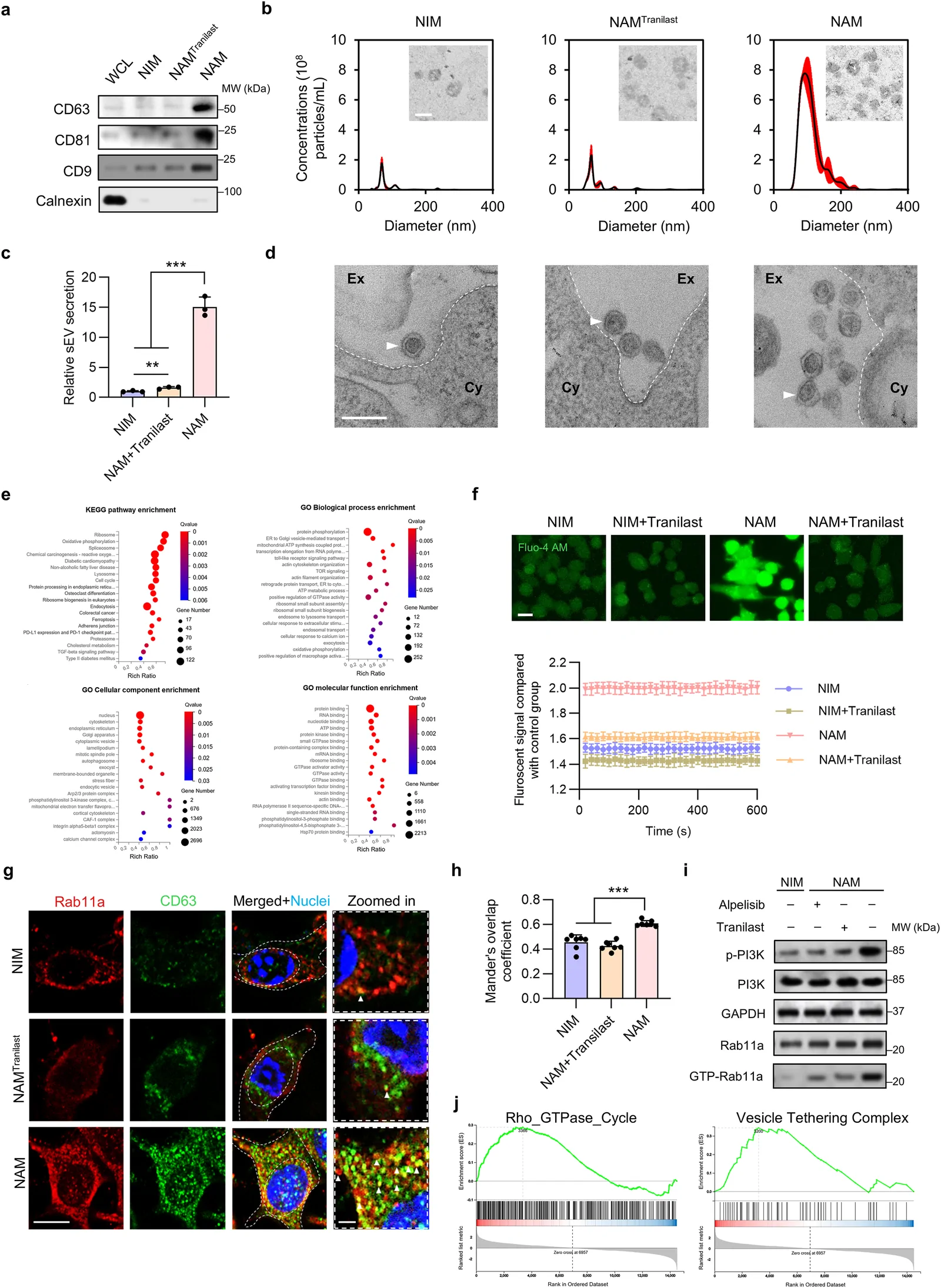
**Matrix nanoalignment enhances TAM-derived sEV secretion through activation of TRPV2/PI3K signaling.** (a) Western blot analysis of whole-cell lysates (WCLs) and isolated sEVs from TAMs primed on NIM or NAM, with or without the TRPV2 inhibitor tranilast. (b) Representative nanoparticle tracking analysis (NTA) profiles of isolated sEVs. Insets show TEM images of collected sEVs from the corresponding groups. Scale bar: 100 nm. (c) Quantification of sEV secretion. sEV concentrations were normalized to the number of viable adherent donor cells and then expressed relative to the NIM group (set to 1; n = 3). (d) Representative TEM images showing vesicles released from the plasma membrane, indicated by white arrows. Ex: extracellular; Cy: cytoplasm. White dotted lines represent cell membrane boundary. Scale bar: 200 nm. (e) KEGG pathway enrichment and GO enrichment analyses of genes upregulated in the NAM group. Bubble size represents gene count; color indicates −log_10_(Q value). (f) Fluo-4 AM staining images with corresponding quantification of Ca^2+^ influx over time in the NIM and NAM groups with or without tranilast treatment. Scale bars: 20 μm. (g) Immunofluorescence images of Rab11a and CD63 showing their colocalization. Scale bars: 10 μm and 2 μm (magnified). (h) Quantitative analysis of measured colocalization between Rab11a and CD63 by Mander's overlap coefficient with Costes (≥95%), involving at least 10 cells from each of three independently prepared hydrogels. (i) Immunoblot analysis of total and phosphorylated PI3K, and total and GTP-bound Rab11a. (j) GSEA plots showing enrichment of hallmark pathways in NAM-cultured macrophages, including the Rho GTPase cycle and vesicle tethering complex. Data are presented as mean ± SD. Statistical significance was determined by one-way ANOVA with Tukey's multiple-comparison test. **p ≤ 0.01; ***p ≤ 0.001.

To identify signaling programs associated with NAM-enhanced sEV release, we analyzed transcriptomic changes in NAM-versus NIM-primed TAMs. Kyoto Encyclopedia of Genes and Genomes (KEGG) and gene ontology (GO) enrichment analyses revealed coordinated enrichment of pathways associated with protein phosphorylation, actin cytoskeleton organization, cellular response to calcium ion, TOR signaling, positive regulation of GTPase activity, exocytosis, and vesicle-mediated transport (Fig. 3e). At the cellular-component and molecular-function levels, NAM-upregulated genes were enriched in cytoskeletal, actomyosin, PI3K complex, calcium channel, exocyst, endocytic vesicle, small GTPase-binding, and phosphoinositide-binding annotations (Fig. 3e). These signatures suggested that matrix nanoalignment activates an integrated mechanotransduction–trafficking program involving calcium-dependent signaling, PI3K activity, and Rab-associated vesicle transport.

To elucidate the molecular mechanism linking nanoscale ligand alignment to enhanced sEV secretion, we focused on mechanosensitive TRPV channels that play a critical role in modulating sEV biogenesis and secretion by acting as a Ca^2+^ channel and triggering the necessary signaling for multivesicular body (MVB) trafficking and fusion with the plasma membrane [[53], [54], [55]]. Among the TRPV family members examined, TRPV2 expression showed the strongest NAM-associated increase (Fig. S14) and was included in the GO term related to calcium-ion transmembrane transport (Table S2). Consistently, NAM-primed TAMs exhibited greater Ca^2+^ influx than NIM-primed TAMs (Fig. 3f). Tranilast markedly attenuated NAM-induced Ca^2+^ influx and reduced sEV release by approximately ninefold, while exerting a comparatively limited effect under NIM conditions (Fig. 3a–d,f). To address the limited specificity of tranilast, we further performed small interfering RNA (siRNA)-mediated TRPV2 knockdown, which similarly reduced NAM-induced Ca^2+^ signaling and sEV release, supporting TRPV2 as an important contributor to the nanoalignment-induced secretory response (Fig. S15).

Importantly, integrin β1 blockade reduced membrane-associated TRPV2 signal, Ca^2+^ influx, and sEV release, placing integrin-dependent matrix sensing functionally upstream of TRPV2-associated signaling (Fig. S16a–c). The alignment-dependent increases in Ca^2+^ signaling and sEV release were also reproduced in collagen I-presenting matrices, indicating that the response was not restricted to cRGD-mediated adhesion (Fig. S16d–f). In contrast, exposure of matrices containing magnetically nonresponsive nanorods to the same magnetic field did not reproduce the NAM-associated increases in TRPV2 clustering, Ca^2+^ influx, or sEV release (Fig. S17), indicating that magnetic-field exposure alone was insufficient to induce these responses. Furthermore, intracellular Ca^2+^ chelation with BAPTA-AM reduced PI3K phosphorylation, whereas both BAPTA-AM treatment and TRPV2 knockdown attenuated calmodulin-dependent protein kinase II (CaMKII) and proline-rich tyrosine kinase 2 (PYK2) phosphorylation (Fig. S18). Collectively, these findings support a functional integrin β1–TRPV2–Ca^2+^–PI3K signaling cascade and identify CaMKII and PYK2 as candidate Ca^2+^-responsive intermediates.

Transcriptomic analysis identified Rab11a as one of the most significantly upregulated Rab GTPases in NAM-primed TAMs, with a strong association to GO terms related to positive regulation of GTPase activity (GO:0043547) and positive regulation of exocytosis (GO:0045921) (Table S2). Immunofluorescence imaging revealed pronounced colocalization of Rab11a with the EV marker CD63 in NAM-primed TAMs, as quantified by Manders’ overlap coefficient (Fig. 3g and h). In contrast, NIM-primed TAMs exhibited weaker CD63 signals and diffuse cytosolic Rab11a distribution. Importantly, NAM priming significantly increased the abundance of GTP-bound active Rab11a (GTP-Rab11a) (Fig. 3i), indicating functional activation compared to those in the NIM group. Gene Set Enrichment Analysis (GSEA) plots supported the enrichment of the Rho GTPase cycle and vesicle tethering complex (Fig. 3j). Pharmacological blocking of TRPV2 or its downstream PI3K activation disrupted the colocalization between CD63 and Rab11a, or reduced GTP-Rab11a (Fig. 3g–i). siRNA-mediated knockdown of Rab11a markedly reduced sEV secretion from NAM-primed TAMs while exerting minimal effects on NIM-cultured cells, whereas Rab11b knockdown produced only a modest reduction (Fig. S19). Rab11a knockdown and PI3K inhibition further induced perinuclear accumulation of CD63 in NAM-primed TAMs (Fig. S19 and Fig. S20), consistent with impaired vesicle trafficking.

### 2.5 NAM priming alters sEV cytokine and miRNA cargo through TRPV2/PI3K–mTORC2-associated signaling

Given the pronounced increase in sEV secretion induced by NAM priming, we next examined whether these sEVs carried functionally distinct molecular cargo [56]. sEVs harvested from NIM- or NAM-primed TAMs (hereafter referred to as sEV^NIM^ and sEV^NAM^, respectively) were analyzed for encapsulated molecules. While sEV^NIM^ contained mixed inflammatory and anti-inflammatory mediators, sEV^NAM^ displayed a striking enrichment of anti-inflammatory cytokines, including IL-10, TGF-β, and vascular endothelial growth factor A (VEGF-A), accompanied by a concomitant reduction in pro-inflammatory cytokines such as TNF-α and IL-6 (Fig. 4a and b). Liquid chromatography-tandem mass spectrometry (LC–MS/MS) profiling further demonstrated distinct protein-cargo compositions in sEV^NIM^​ and sEV^NAM^​ (Fig. S21a). sEV^NIM^​ contained higher levels of representative pro-inflammatory proteins, whereas sEV^NAM^​ was enriched in selected immunoregulatory and tissue-remodeling proteins (Fig. S21b and c). GO-based GSEA further indicated reduced type II interferon-associated responses and increased nucleic-acid metabolism and protein-modification pathways in sEV^NAM^ (Fig. S21d). Small RNA sequencing further revealed a distinct miRNA signature associated with NAM priming. A total of 47 miRNAs were significantly upregulated and 32 miRNAs downregulated in sEV^NAM^ relative to sEV^NIM^ (Q < 0.05) (Fig. 4c). Among these, miR-181a-5p showed prominent particle-normalized enrichment in sEV^NAM^ relative to sEV^NIM^ (Fig. 4d and e). Because the cytokine and miRNA analyses were performed using equal numbers of sEV particles across groups, these findings indicate that nanoscale ECM alignment not only increases total sEV output but also alters the particle-normalized cargo composition of the resulting sEV population. Accordingly, the increased abundance of miR-181a-5p in sEV^NAM^ cannot be explained solely by the greater number of sEVs released under NAM conditions.

**Fig. 4:**
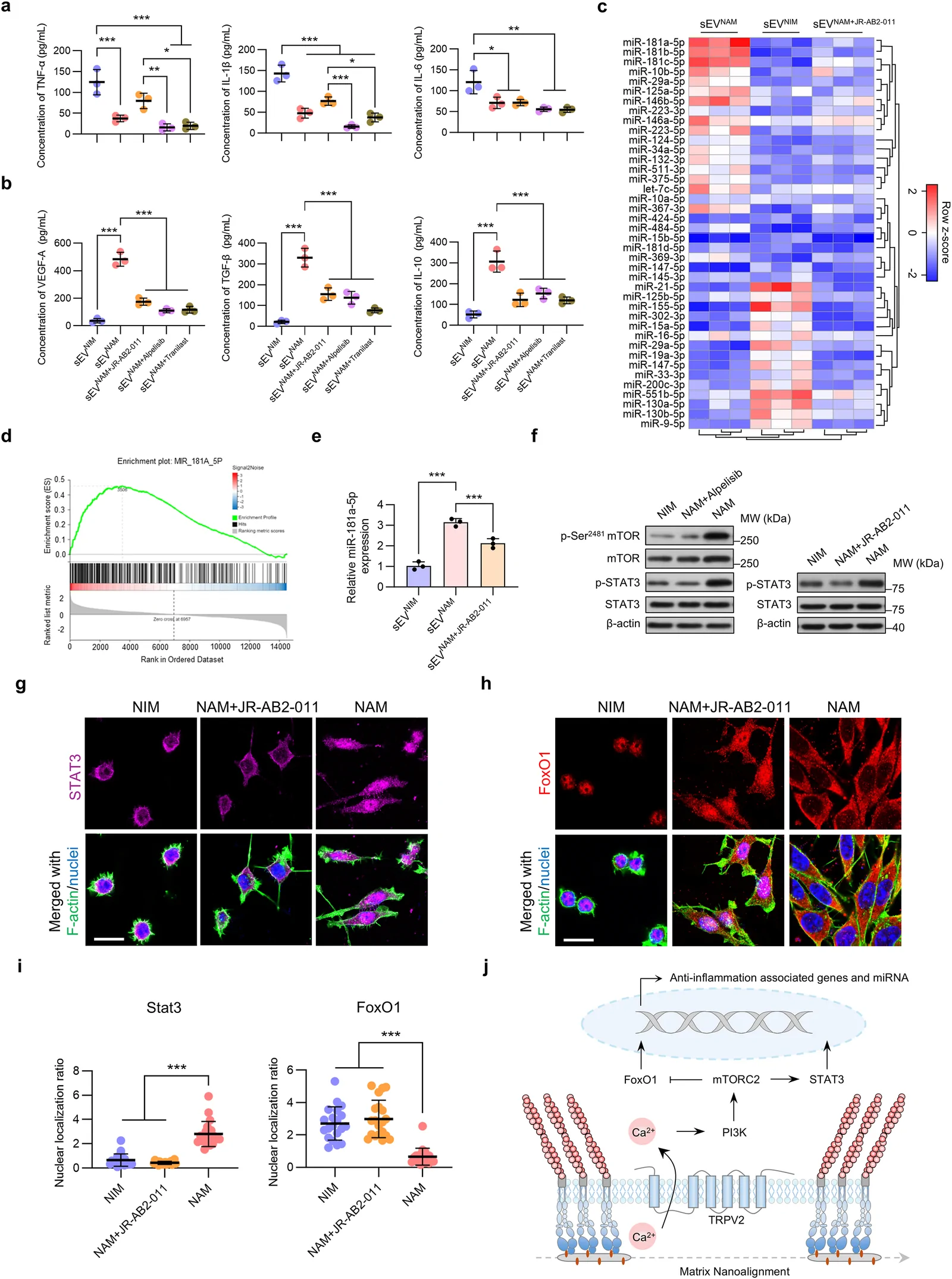
**Nanoscale alignment alters TAM-derived sEV cargo through TRPV2/PI3K–mTORC2-associated signaling.** (a,b) ELISA quantification of pro-inflammatory (a) and anti-inflammatory (b) cytokines in sEVs isolated from NIM- or NAM-primed TAMs. (c) Differential miRNA profiling of sEVs from NIM (sEV^NIM^), NAM (sEV^NAM^), or NAM plus mTORC2 inhibitor (sEV^NAM + JR−AB2-011^) groups (n = 3; Q < 0.05). (d) GSEA highlighting miR-181a-5p-associated pathways enriched in sEV^NAM^ versus sEV^NIM^. (e) Particle-normalized qPCR validation of miR-181a-5p abundance in the indicated sEV groups (n = 3). (f) Immunoblot analysis of mTOR Ser2481 and STAT3 phosphorylation. (g,h) Immunofluorescence staining of (g) STAT3 and (h) FoxO1, with (i) quantification of nuclear localization ratios (n = 20 cells from three independent gels). Scale bar: 20 μm. (j) Proposed molecular mechanism illustrating NAM-induced upregulation of anti-inflammatory genes and miRNAs via PI3K–mTORC2-associated signaling. Data are presented as mean ± SD. Statistical significance was determined by one-way ANOVA with Tukey's multiple-comparison test. *p ≤ 0.05; **p ≤ 0.01; ***p ≤ 0.001.

To explore signaling mechanisms associated with the increased miR-181a-5p abundance in sEV^NAM^, we focused on the PI3K/Akt–mTOR pathway, which was strongly enriched in transcriptomic and GSEA analyses (Fig. 3e). Immunoblotting revealed increased mTOR Ser2481 phosphorylation in NAM-primed TAMs (Fig. 4f), a phosphorylation event commonly used as an mTOR complex 2 (mTORC2)-associated signaling readout, suggesting enhanced mTORC2 signaling activity. In parallel, NAM priming induced robust phosphorylation and nuclear accumulation of STAT3, accompanied by cytoplasmic exclusion of FoxO1 (Fig. 4g–i). STAT3 has been reported to transcriptionally activate miR-181 family members, either directly or through cooperation with SMAD and AP-1 transcriptional complexes [57,58], whereas FoxO1 maintains chromatin accessibility of inflammatory gene loci [59]. Consistent with this regulatory framework, pharmacological inhibition of mTORC2 reduced miR-181a-5p abundance in sEV^NAM^, and was accompanied by reduced STAT3 nuclear localization and increased FoxO1 nuclear retention in the donor TAMs (Fig. 4g–i). Inhibition of TRPV2, PI3K, or mTORC2 during NAM priming also reduced anti-inflammatory cytokine abundance and miR-181a-5p levels in the resulting sEVs (Fig. 4a–c,e). Together, these results support a model in which nanoscale ligand alignment-induced TRPV2–PI3K signaling engages an mTORC2-associated STAT3/FoxO1 regulatory program that contributes to the altered immunoregulatory cargo profile of sEV^NAM^ (Fig. 4j). Because STAT3 and FoxO1 were not independently perturbed in the present study, their precise causal contributions to miR-181a-5p regulation remain to be established. Reciprocal genetic perturbation further showed that Rab11a depletion did not alter mTOR Ser2481 phosphorylation (Fig. S22a), whereas Rictor-targeting siRNA treatment did not significantly alter GTP-bound Rab11a (Fig. S22b), supporting partially separable PI3K–Rab11a and PI3K–mTORC2-associated outputs.

### 2.6 miR-181a-5p-enriched sEVs suppress TLR4–NF–κB signaling and reinforce the anti-inflammatory phenotype of macrophages

To directly assess the functional impact of sEV^NIM^ and sEV^NAM^ on macrophage polarization, naïve macrophages were treated with the respective sEVs in the presence of pro-inflammatory or anti-inflammatory mediators. sEV^NAM^ markedly promoted macrophage proliferation and induced M2-like polarization against inflammatory conditions, whereas sEV^NIM^ exerted minimal effects (Fig. 5a,b and S23–24). These effects were significantly attenuated when sEV^NAM^ was generated in the presence of the mTORC2 inhibitor JR-AB2-011 (Fig. 5a and b), supporting a contribution of mTORC2-associated cargo remodeling to the immunoregulatory activity of sEV^NAM^.

**Fig. 5:**
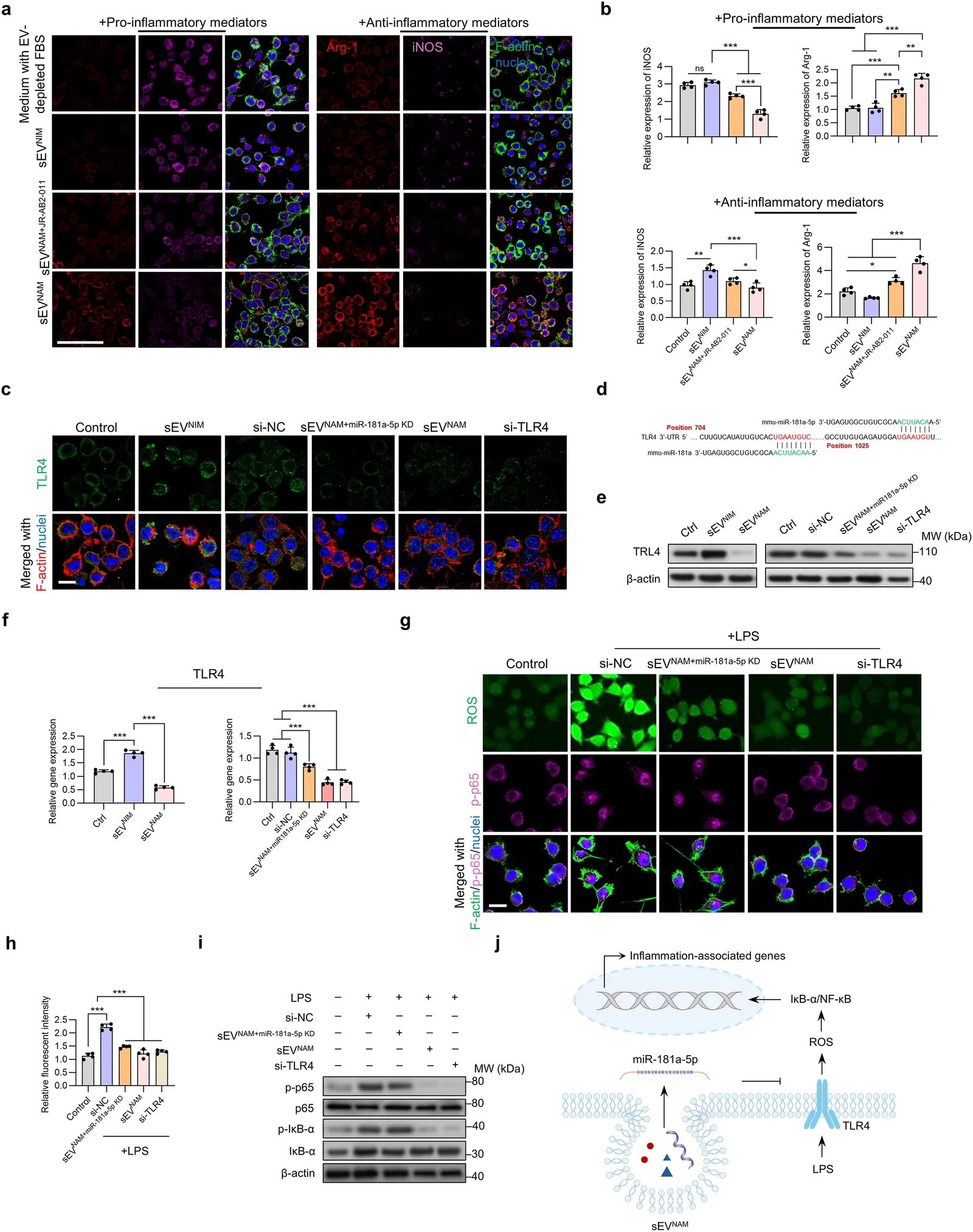
**NAM-primed TAM-derived sEVs are enriched in miR-181a-5p and suppress TLR4-mediated inflammatory signaling in macrophages.** (a) Confocal immunofluorescence images of iNOS and Arg-1 staining after 36 h of sEV treatment, with (b) corresponding gene expression quantification under stimulation with pro-inflammatory or anti-inflammatory mediators, respectively. (c) Immunofluorescence images of TLR4. (d) Predicted alignment of miR-181a-5p with the TLR4 3′-UTR based on TargetScan and miRanda analyses. (e) Protein quantification of TLR4 and (f) corresponding gene expression analysis. (g) Confocal images of intracellular ROS and phosphorylated NF-κB (p-p65), with (h) ROS intensity quantification. (i) Immunoblot quantification of IκB-α and NF-κB phosphorylation. (j) Proposed mechanism illustrating miR-181a-5p-mediated suppression of TLR4 signaling and inflammatory gene expression. Groups include TAMs treated with blank control, scrambled TLR4 siRNA (si-NC), sEV^NAM^ with miR-181a-5p knockdown (sEV^NAM + miR−181a−5p KD^), sEV^NAM^, or TLR4 siRNA (si-TLR4). Data are presented as mean ± SD (n = 4 independent biological experiments). Statistical significance was determined by one-way ANOVA with Tukey's multiple-comparison test. *p ≤ 0.05; **p ≤ 0.01; ***p ≤ 0.001.

Mechanistically, sEV^NAM^ treatment substantially downregulated TLR4 expression at both the mRNA and protein levels (Fig. 5c–e,f). Bioinformatic analysis identified two conserved miR-181a-5p binding sites within the 3′ untranslated region of TLR4 (Fig. 5d). Suppression of TLR4 signaling translated into reduced reactive oxygen species generation, diminished phosphorylation of NF-κB p65, and stabilization of IκB-α (Fig. 5g–i). These effects were attenuated when miR-181a-5p was depleted from sEV^NAM^ and were phenocopied by TLR4 knockdown, supporting a functional contribution of the miR-181a-5p–TLR4 axis to sEV^NAM^-mediated suppression of inflammatory signaling. As sEV^NAM^ was also simultaneously enriched in IL-10 and TGF-β proteins (Fig. 4b), these could participate in M2 polarization of macrophages [44,60,61]. IL-10 neutralization or TGF-β receptor inhibition partially reduced sEV^NAM^-induced Arg-1 expression and increased iNOS expression, whereas combined treatment with miR-181a-5p depletion produced a stronger reversal (Fig. S25). These findings suggest cooperative contributions of miR-181a-5p and IL-10/TGF-β-associated signaling. Together, these findings indicate that sEV^NAM^ acts through multiple cargo components. miR-181a-5p primarily contributes to suppression of the TLR4–NF-κB inflammatory pathway (Fig. 5j).

### 2.7 NAM-primed TAM-derived sEVs exacerbate melanoma progression and immunosuppression in vivo

We first explored whether sEV^NAM^ influenced M1-like/M2-like macrophage balance in vivo. To achieve this, we performed subcutaneous injection of phosphate-buffered saline (PBS as control), sEV^NIM^, sEV^NAM^ or sEV^NAM + miR−181a−5p KD^ in 6-week-old BALB/c mice at 3-day intervals over 14 days (Fig. S26a). Skin surrounding the injection site was harvested and stained for CD68/CD86 and CD68/CD206 to identify dermal M1-like and M2-like macrophages, respectively, following an established protocol [62]. Our data showed that subcutaneous injection of sEV^NAM^ skewed macrophage populations toward increased M2-like macrophage abundance with a higher proportion (Fig. S26b and c), and enhanced collagen deposition in skin tissue (Fig. S27a and b). In addition, the dermal layer was thicker in the sEV^NAM^ group than in the other groups, increasing to approximately 239 μm compared with approximately 200 μm in the PBS group (Fig. S27c). These findings indicate that sEV^NAM^ can modulate macrophage balance and influence ECM deposition in vivo.

We next examined the functional consequences of NAM-primed TAM-derived sEVs in a syngeneic melanoma model (Fig. 6a). In vivo and ex vivo imaging of fluorescently labeled sEVs demonstrated detectable tumor accumulation within 48 h despite predominant hepatic and splenic clearance post intravenous injection of the sEVs (Fig. S28). Tumor-section imaging further showed colocalization of the sEV-associated fluorescence with CD68^+^CD163^+^ TAMs (Fig. S29). Subsequently, sEV^NAM^ markedly accelerated tumor growth compared with sEV^NIM^-treated or PBS-treated controls, as evidenced by increased tumor volume over a 30-day period (Fig. 6b and c). Histological analyses further revealed enhanced tumor cell proliferation, as demonstrated by increased EdU incorporation, alongside elevated α-SMA and CD31 staining, indicating augmented stromal activation and angiogenesis in sEV^NAM^-treated tumors (Fig. 6b and c).

**Fig. 6:**
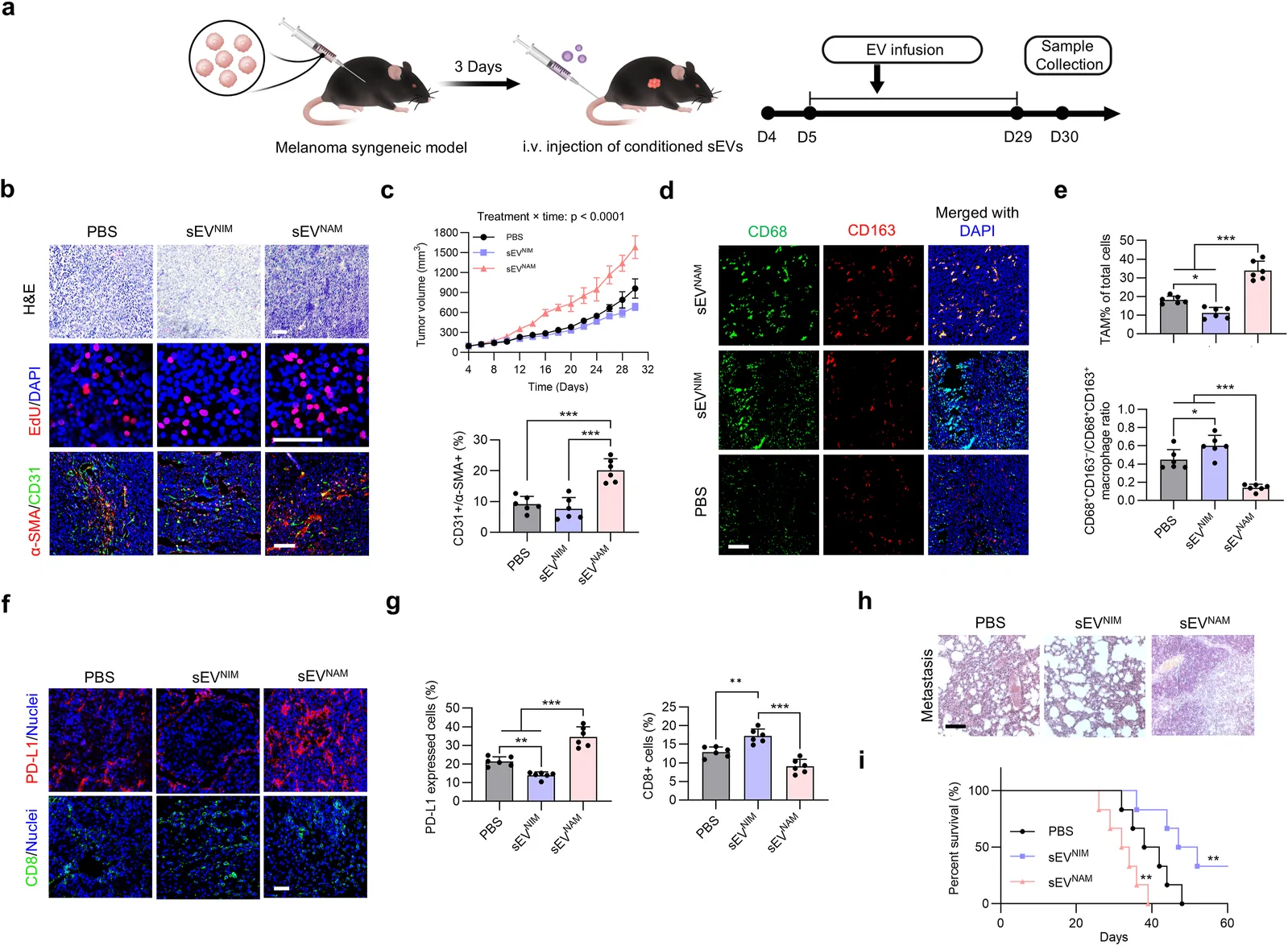
**sEV^NAM^ promotes melanoma growth and aggressiveness in vivo.** (a) Schematic of the syngeneic melanoma model and intravenous sEV administration schedule. Conditioned sEVs were administered every 3 days, and samples were collected on day 30. (b) Representative H&E staining, EdU proliferation staining, and α-SMA/CD31 immunofluorescence of tumor tissues from the PBS, sEV^NIM^, and sEV^NAM^ groups. (c) Longitudinal tumor growth curves and quantification of CD31^+^α-SMA^+^ vessels. (d) Representative CD68/CD163 immunofluorescence staining of tumor tissues. (e) Quantification of TAM abundance and CD163^+^ TAM proportion. (f) Representative immunofluorescence staining of PD-L1 and CD8 in tumor tissues. (g) Quantification of PD-L1^+^ and CD8^+^ cells. (h) Representative H&E staining of lung tissues showing metastatic lesions. (i) Kaplan–Meier survival curves (log-rank test). Scale bars: 100 μm in (b), (d), and (f); 200 μm in (h). Data are presented as mean ± SD (n = 6 independent mice). Tumor growth curves in (c) were analyzed by two-way repeated-measures ANOVA, with treatment as the between-subject factor and time as the within-subject factor, using the Geisser–Greenhouse correction, followed by Tukey's multiple-comparison test for between-group comparisons at individual time points. Other endpoint comparisons in (c), (e), and (g) were analyzed by one-way ANOVA followed by Tukey's multiple-comparison test. *p ≤ 0.05, **p ≤ 0.01, and ***p ≤ 0.001.

Consistent with these macroscopic observations, immunofluorescence staining of tumor sections revealed a substantial increase in CD68^+^CD163^+^ TAM infiltration in sEV^NAM^-treated tumors, accompanied by a pronounced shift in the macrophage phenotype proportion toward an M2-dominant phenotype (Fig. 6d and e). This macrophage reprogramming was paralleled by elevated PD-L1 expression within the tumor parenchyma and a significant reduction in CD8^+^ cytotoxic T-cell infiltration (Fig. 6f and g). Consistently, KEGG enrichment analysis of the miR-181a-5p-responsive gene set highlighted pathways related to PD-L1 expression and PD-1 checkpoint signaling, together with other tumor-promoting pathways such as TGF-β signaling (Fig. S30). Kaplan–Meier survival analysis further demonstrated that mice receiving sEV^NAM^ exhibited significantly shorter survival compared with control groups (Fig. 6h and i), underscoring the pathological impact of NAM-primed TAM-derived sEVs on tumor progression. These in vivo findings directly corroborate our in vitro mechanistic data, demonstrating that sEVs generated under nanoscale-aligned ECM conditions are not merely phenotypic markers of TAM polarization but functional mediators of immune suppression and tumor growth.

### 2.8 Targeted inhibition of TAM-associated sEV release alleviates immunosuppression in vivo

We next investigated whether selective disruption of TAM-derived sEV release could therapeutically reverse the immunosuppressive tumor microenvironment. To achieve cell-type-preferential intervention [[63], [64], [65]], we engineered M2 macrophage-targeting peptide (M2pep)-modified lipid nanocarriers loaded with tranilast to inhibit TRPV2 signaling, Rab11a siRNA (Rab11a-siRNA) to suppress Rab11a-dependent vesicle trafficking, or galunisertib to inhibit TGF-β signaling and attenuate matrix remodeling, respectively (Fig. 7a). Empty lipid nanocarriers served as vehicle controls. This strategy enabled parallel targeting of the macrophage-intrinsic TRPV2–Rab11a sEV-release pathway and the TGF-β-associated extracellular matrix remodeling program that may contribute to the immunosuppressive tumor niche [5]. Flow-cytometric analysis of dissociated tumors confirmed preferential, although not exclusive, uptake of fluorescently labeled M2pep-modified lipid nanocarriers by CD206^+^ TAMs (Fig. S31), supporting TAM-oriented delivery in vivo.

**Fig. 7:**
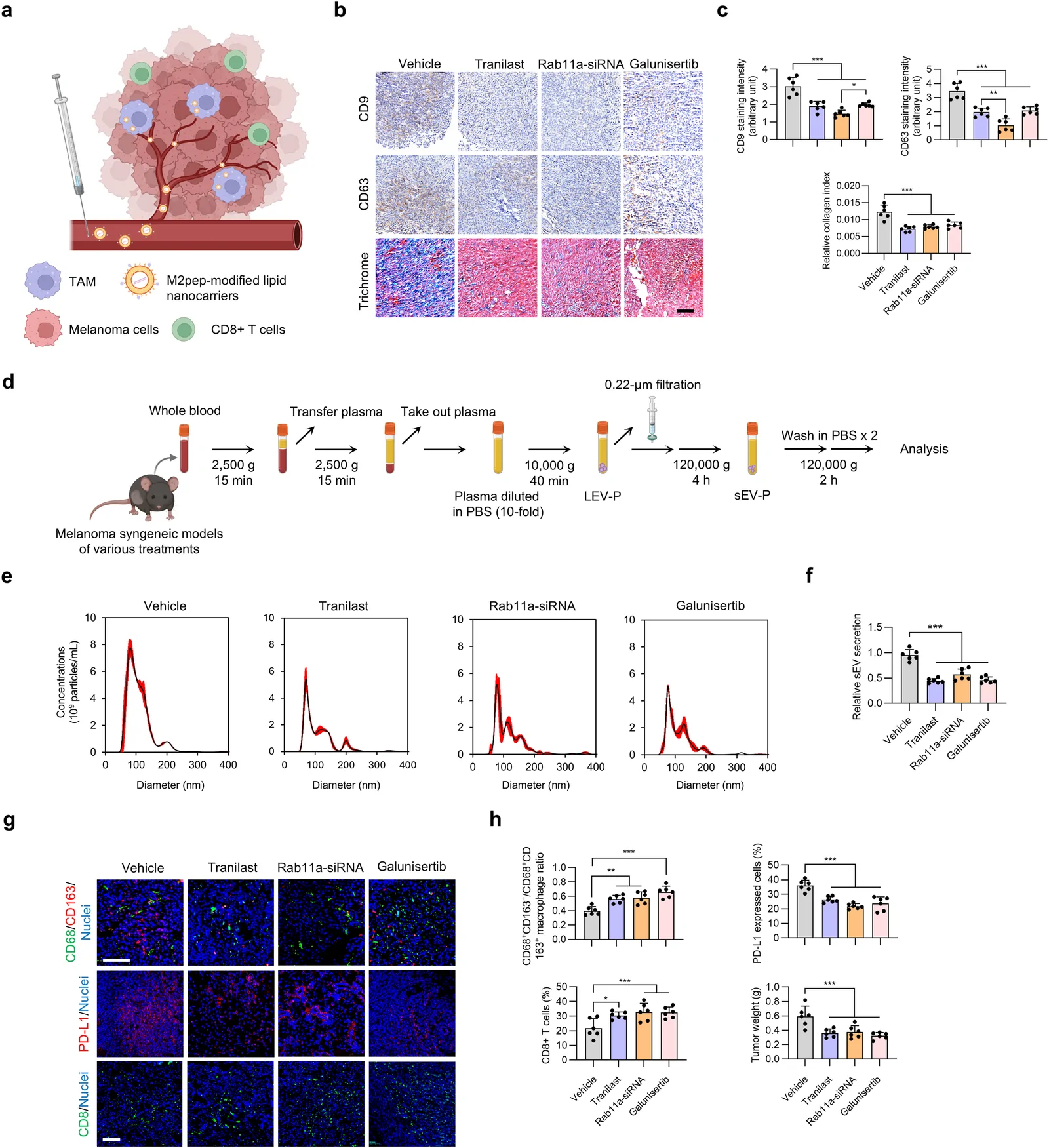
**Targeted inhibition of TAM mechanosensitive sEV programming reduces circulating sEV abundance and alleviates melanoma-associated immunosuppression in vivo.** (a) Schematic illustration of intravenous administration of M2 macrophage-targeting peptide (M2pep)-modified lipid nanocarriers loaded with tranilast, galunisertib, or Rab11a-siRNA in the syngeneic melanoma model. (b) Representative immunohistochemical staining for EV markers CD9 and CD63 and Masson's trichrome staining of tumor sections from different treatment groups. (c) Quantification of CD9/CD63-positive staining intensity and Masson's trichrome-positive collagen deposition. (d) Schematic illustration of plasma-derived sEV isolation from melanoma-bearing mice following the indicated treatments. (e) Representative NTA profiles of plasma-derived sEVs. (f) Quantification of circulating sEV concentration. sEV concentrations were normalized to the vehicle group (set to 1). (g) Representative immunofluorescence staining of CD68/CD163, PD-L1, and CD8 in tumor tissues. (h) Quantification of the CD68^+^CD163^+^/CD68^+^CD163^-^ macrophage ratio, PD-L1^+^ cells, CD8^+^ T-cell infiltration, and tumor weight. Scale bars: 100 μm. Data are presented as mean ± SD (n = 6 independent mice). Statistical significance was determined by one-way ANOVA followed by Tukey's multiple-comparison test. *p ≤ 0.05, **p ≤ 0.01, and ***p ≤ 0.001. Schematics in (a) and (d) were created with BioRender.com.

Compared with vehicle treatment, CD9 and CD63 signals within tumor tissues were reduced following inhibition of TRPV2, Rab11a, or TGF-β signaling, with the most pronounced reduction observed in the Rab11a-siRNA group (Fig. 7b and c). Consistently, these interventions decreased the concentration of plasma-derived sEVs measured by NTA (Fig. 7d–f), indicating that disruption of either the TRPV2–Rab11a secretory pathway or the TGF-β-associated matrix-remodeling program can attenuate systemic TAM-derived sEV release in vivo [66]. Importantly, these treatments also reduced collagen deposition within tumors (Fig. 7b and c), suggesting that modulation of TAM secretory activity may influence the reciprocal relationship between extracellular matrix remodeling and immune suppression.

Finally, targeting these pathways increased the CD68^+^CD163^+^/CD68^+^CD163^-^ macrophage ratio, reduced PD-L1-positive cells, and enhanced CD8^+^ T-cell infiltration, resulting in decreased tumor growth and tumor weight compared with vehicle treatment (Fig. 7g and h), consistent with previous reports [67,68]. These immunological changes were accompanied by reduced tumor burden, demonstrating that targeting TAM-associated sEV communication represents an effective strategy to alleviate nanoalignment-associated tumor immune evasion.

## 3 Conclusion

Our findings establish ECM nanoarchitecture as an active regulator of macrophage immune communication. By decoupling nanoscale ligand alignment from bulk matrix mechanics, the NAM/NIM platform demonstrates that adhesive nanoalignment is sufficient to enhance integrin β1-dependent mechanosensing and TRPV2-mediated Ca^2+^ signaling in TAMs. Downstream PI3K signaling bifurcates into a Rab11a-dependent branch that controls sEV trafficking and release and an mTORC2-associated branch that contributes to immunoregulatory cargo remodeling (Fig. 8). Together, these separable effects on vesicle output and particle-normalized cargo composition generate an sEV population preferentially enriched in anti-inflammatory cytokines, miR-181a-5p, and selected tissue-remodeling proteins, thereby suppressing TLR4–NF-κB signaling, reinforcing M2-like macrophage states, limiting CD8^+^ T-cell infiltration, and accelerating melanoma progression.

**Fig. 8:**
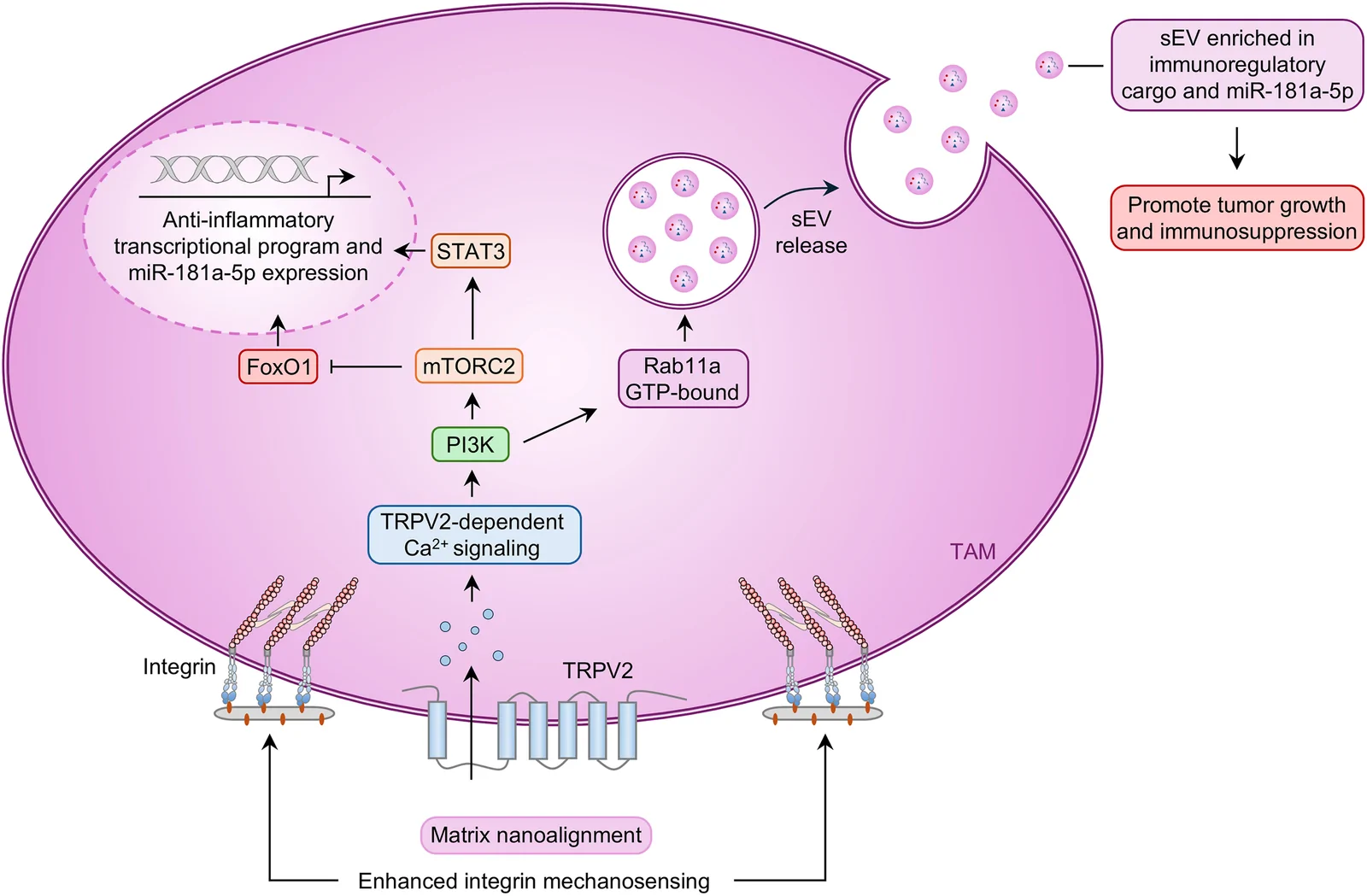
**Proposed model of matrix nanoalignment-induced TAM sEV release and immunoregulatory cargo remodeling.** Matrix nanoalignment enhances integrin-mediated mechanosensing and activates TRPV2-dependent Ca^2+^ signaling, leading to PI3K activation. PI3K promotes accumulation of GTP-bound Rab11a, facilitating multivesicular body trafficking and fusion with the plasma membrane and thereby increasing sEV release. In parallel, PI3K–mTORC2-associated signaling is accompanied by STAT3 activation and reduced FoxO1-associated inflammatory activity, together contributing to anti-inflammatory gene expression and enrichment of miR-181a-5p and other immunoregulatory cargo in sEVs. These NAM-primed sEVs subsequently promote an immunosuppressive tumor microenvironment and tumor progression. Arrows summarize functional regulatory relationships supported by the present data and do not necessarily indicate direct physical interactions between the indicated molecules.

These results advance the field in three respects. First, they identify nanoscale ECM alignment as a distinct physical cue capable of regulating macrophage secretory behavior independently of bulk stiffness. Second, they define separable Rab11a- and mTORC2-associated outputs downstream of a common mechanosensitive PI3K node, corresponding to sEV release and cargo remodeling, respectively. Third, they show that preferential intervention in the TRPV2–Rab11a pathway or TGF-β-associated matrix remodeling can reduce circulating sEV abundance, alleviate tumor immunosuppression, and restrict melanoma growth.

The NAM/NIM platform is a reductionist cRGD-presenting system and does not reproduce the full compositional, degradable, viscoelastic, and three-dimensional complexity of native melanoma stroma [69]. Decellularized tumor ECM and self-assembled collagen matrices more closely retain selected aspects of this native complexity and have demonstrated that tumor-derived ECM can directly educate macrophage phenotypes [70]. Accordingly, the magnitude of the observed response, including the approximately tenfold increase in sEV output, should not be assumed to be universal across all ECM contexts. Nevertheless, validation with collagen-presenting matrices, genetic perturbation, proteomic cargo profiling, and in vivo intervention supports a robust link between matrix nanoalignment and TAM vesicular immunity. Together, this work provides a mechanistic framework connecting nanoscale adhesive organization to immunosuppressive intercellular communication and identifies TAM mechanotransduction as a potential therapeutic vulnerability in matrix-remodeled, macrophage-rich tumors.

## CRediT authorship contribution statement

**Bohan Yin:** Writing – review & editing, Writing – original draft, Visualization, Supervision, Methodology, Investigation, Funding acquisition, Formal analysis, Data curation, Conceptualization. **Guangli Xiang:** Writing – original draft, Visualization, Formal analysis, Data curation, Methodology, Investigation. **Ziqi Zhang:** Methodology, Investigation, Formal analysis, Data curation. **Limeng Zheng:** Methodology, Investigation, Formal analysis, Data curation. **Runyao Wang:** Writing – original draft, Visualization, Formal analysis, Data curation. **Qin Zhang:** Investigation, Data curation. **Yingying Huang:** Validation, Formal analysis, Data curation. **Chenjie Liu:** Methodology, Investigation, Data curation. **Jingjing Chi:** Methodology, Formal analysis, Data curation. **Xinyue Xia:** Software, Formal analysis, Data curation. **Jiaxiang Yan:** Formal analysis, Data curation. **Jianfang Wang:** Investigation, Formal analysis. **Mo Yang:** Validation, Methodology, Investigation. **Liming Bian:** Writing – review & editing, Writing – original draft, Supervision, Investigation. **Siu Hong Dexter Wong:** Writing – review & editing, Writing – original draft, Visualization, Validation, Supervision, Resources, Project administration, Methodology, Investigation, Funding acquisition, Formal analysis, Data curation, Conceptualization.

## Ethics approval and consent to participate

All animal procedures were approved by the Scientific Ethics Committee of the Academic Committee of Ocean University of China (OUC-AE-2024-091 and OUC-AE-2026-205). This study does not involve human subjects.

## Declaration of competing interest

The authors have a Chinese patent application related to this work with application no. 2025115102059 and have noted the patents of immediate family members.
