# Corrigendum to “Matrix nanoalignment programs macrophage vesicular immunity through TRPV2 mechanogating” [Bioact. Mater. 69 (2027) 18–32]

**DOI:** 10.1016/j.bioactmat.2026.10.018

**Published:** 2026-10-15

**Authors:** Bohan Yin, Guangli Xiang, Ziqi Zhang, Limeng Zheng, Runyao Wang, Qin Zhang, Yingying Huang, Chenjie Liu, Jingjing Chi, Xinyue Xia, Jiaxiang Yan, Jianfang Wang, Mo Yang, Liming Bian, Siu Hong Dexter Wong

**Affiliations:** a School of Medicine and Pharmacy, Ocean University of China, Qingdao, 266100, China; b Laboratory for Marine Drugs and Bioproducts, Qingdao Marine Science and Technology Center, Qingdao, 266237, China; c Department of Biomedical Engineering, The Hong Kong Polytechnic University, Hong Kong, 999077, China; d School of Biomedical Sciences and Engineering, South China University of Technology, Guangzhou International Campus, Guangzhou, 511442, China; e Department of Physics, The Chinese University of Hong Kong, Hong Kong, 999077, China

The authors regret that, during figure assembly, several representative microscopy panels in Figs. 5c and 7g required correction due to inadvertent field selection and image overlap. Following re-examination of the original image files and figure-assembly records, the affected panels were identified and the verified images for replacement were retrieved from the corresponding experimental records. The published panels in Figs. 5a and 6f were confirmed against their original source files to be correctly assigned and therefore remain unchanged. For Fig. 7g, the complete PD-L1/nuclei row has been replaced to ensure consistent magnification and presentation across treatment groups.

1. **Fig. 5c.** The Control and si-NC representative fields in Fig. 5c were inadvertently mis-selected. Consequently, the published Control TLR4 image overlapped with Fig. 5a Arg-1 image for the “Medium with EV-depleted FBS” group under anti-inflammatory conditions, while the published si-NC TLR4 image and its corresponding merged F-actin/nuclei view overlapped with Fig. 5a sEV^NAM^ field under pro-inflammatory conditions. Review of the original source files confirmed that the corresponding Fig. 5a images are correctly assigned and are retained unchanged. In the corrected Fig. 5, Fig. 5c Control and si-NC representative fields, including their corresponding merged views, have been replaced with verified images corresponding to their stated experimental conditions.
2. **Fig. 7g.** The published Tranilast PD-L1/nuclei representative field in Fig. 7g was inadvertently mis-selected and overlapped with the correctly assigned sEV^NAM^ CD8/nuclei representative image in Fig. 6f. Review of the original source files confirmed that Fig. 6f image is correctly assigned and therefore remains unchanged. During the expanded internal review of Fig. 7g, the authors also identified that the published Galunisertib PD-L1/nuclei panel partially overlapped with the Vehicle field. The published Vehicle PD-L1/nuclei panel was verified as correctly assigned. To ensure consistent magnification and presentation across the corrected comparison, the entire PD-L1/nuclei row in Fig. 7g (Vehicle, Tranilast, Rab11a-siRNA, and Galunisertib) has been replaced with verified, independent representative fields corresponding to the respective treatment groups.

The authors have re-examined the original image datasets and the corresponding quantitative analyses for these experiments. The corrections described above concern only the selection and presentation of representative microscopy images during figure assembly. The quantitative datasets and statistical analyses were generated from the appropriate experimental groups and remain unchanged. Accordingly, the reported results, interpretation, and conclusions of the article are unaffected.

3. **Figure S26b.** The label of the fourth representative-image column was inadvertently written as “sEV^NIM^”. The correct label is “sEV^NAM^”. The representative image itself is unchanged.
4. **Figure S27a.** The label of the fourth representative-image column was inadvertently written as “sEV^NIM^”. The correct label is “sEV^NAM^”. The representative images themselves are unchanged.

The corrected Figs. 5 and 7 should replace the corresponding published figures. Figs. 5a and 6f require no modification. In Fig. 7g, all four PD-L1/nuclei representative panels have been replaced with verified independent fields to ensure consistent magnification and presentation across treatment groups. The Supporting Information should be updated only to correct the labels in Figures S26b and S27a.

**Figure:**
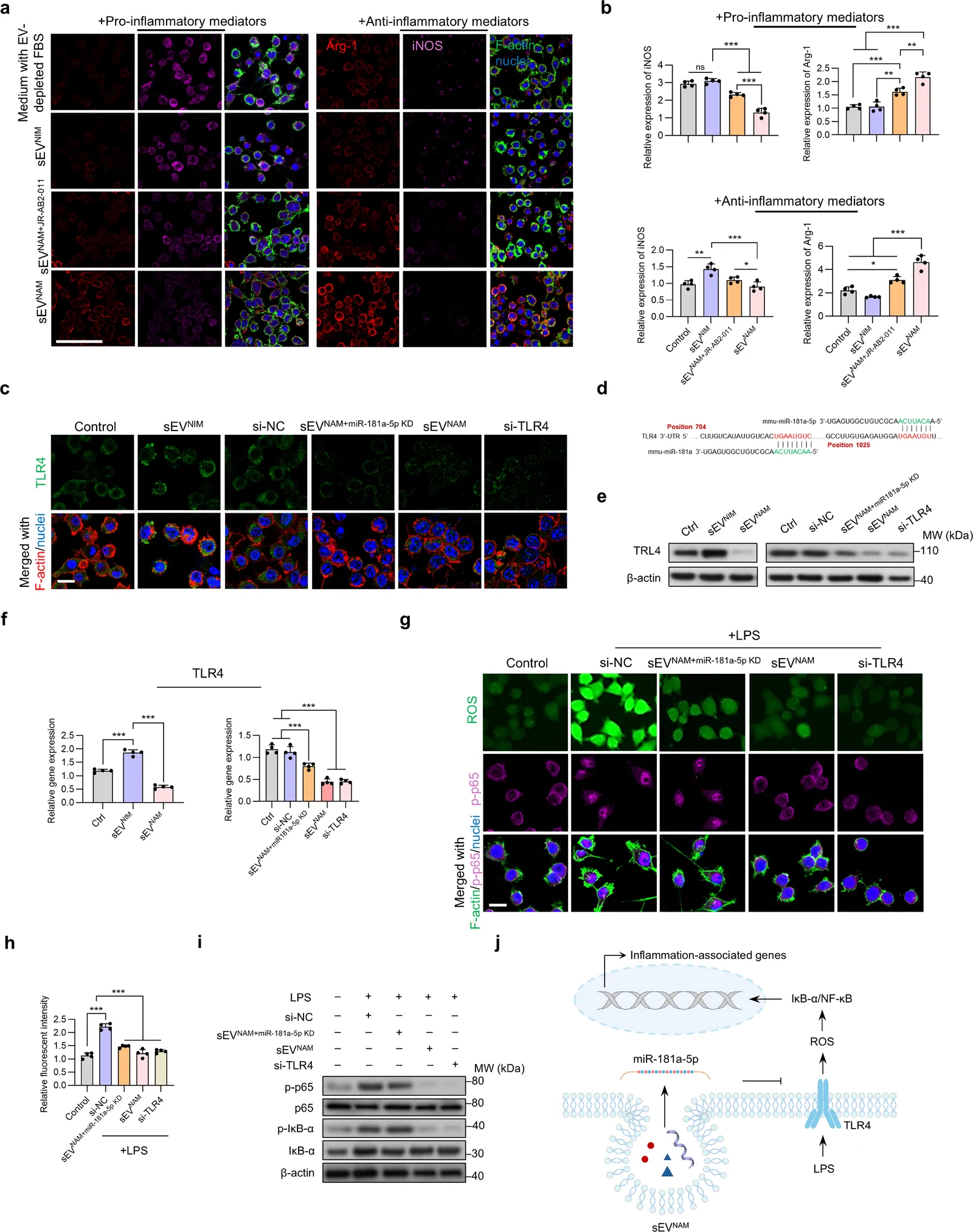

Corrected Fig. 5

**Figure:**
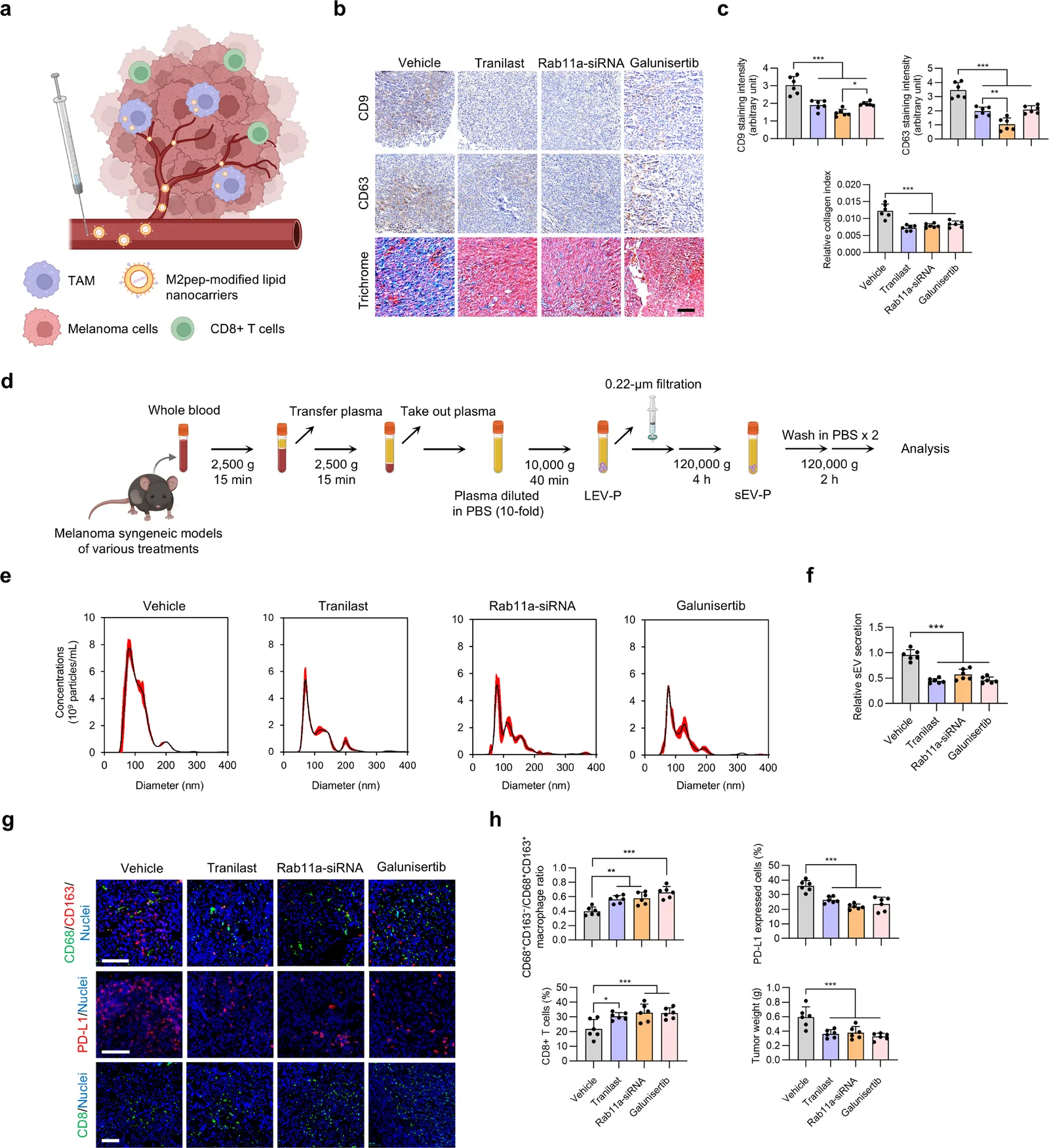

Corrected Fig. 7

The authors would like to apologize for any inconvenience caused.

