# Supplementary material for: Matrix nanoalignment programs macrophage vesicular immunity through TRPV2 mechanogating
Source: Bioact Mater. 2026 Sep 23;69:18–32. doi: 10.1016/j.bioactmat.2026.09.011 (PMC13634591; doi:10.1016/j.bioactmat.2026.09.011)
Supplement: Supplementary file 1 [file mmc1.docx]

**Supporting Information**

**Matrix nanoalignment programs macrophage vesicular immunity through TRPV2 mechanogating**

Bohan Yin,^1,2^ Guangli Xiang,^1,2^ Ziqi Zhang,^1^ Limeng Zheng,^1^ Runyao Wang,^1^ Qin Zhang,^3^ Yingying Huang,^3^ Chenjie Liu,^1^ Jingjing Chi,^1^ Xinyue Xia,^5^ Jiaxiang Yan,^3^ Jianfang Wang,^5^ Mo Yang,^3^ Liming Bian,*^,4^ Siu Hong Dexter Wong^1,2,^*

^1^School of Medicine and Pharmacy, Ocean University of China, Qingdao 266100, China;

^2^Laboratory for Marine Drugs and Bioproducts, Qingdao Marine Science and Technology Center, Qingdao 266237, China;

^3^Department of Biomedical Engineering, The Hong Kong Polytechnic University, Hong Kong 999077, China;

^4^School of Biomedical Sciences and Engineering, South China University of Technology, Guangzhou International Campus, Guangzhou 511442, China;

^5^Department of Physics, The Chinese University of Hong Kong, Hong Kong 999077, China;

**Corresponding authors***

Liming Bian: [bianlm@scut.edu.cn](mailto:bianlm@scut.edu.cn)

Siu Hong Dexter Wong: [dexterwong@ouc.edu.cn](mailto:dexterwong@ouc.edu.cn)

**Keywords:** ECM nanoarchitecture, Tumor-associated macrophages, Mechanobiology, small extracellular vesicle biogenesis, Magnetic Control

**Methods**

All research presented here complies with all relevant ethical regulations.

1. *Materials and reagents*

Sodium hyaluronate (weight-average molecular weight: 40–70 kDa; Lifecore), methacrylic anhydride (Sigma-Aldrich, 276685), Irgacure 2959 (Aladdin, H137984), ferric chloride hexahydrate (FeCl₃·6H₂O; Sigma-Aldrich, 44945), polyacrylic acid (PAA; Sigma-Aldrich, 416002), tetraethyl orthosilicate (TEOS; Sigma-Aldrich, 131903), diethylene glycol (DEG; Sigma-Aldrich, V900241), 3-aminopropyltriethoxysilane (APTES; Sigma-Aldrich, 741442), 4-maleimidobutyric acid N-hydroxysuccinimide ester (Mal-NHS; Sigma-Aldrich, 63133), tris(2-carboxyethyl)phosphine (TCEP; Sigma-Aldrich, 646547), and 5,5′-dithiobis-(2-nitrobenzoic acid) (DTNB; Ellman’s reagent; Sigma-Aldrich, D8130) were purchased from Sigma-Aldrich unless otherwise specified. Osmium tetroxide, propylene oxide, epoxy resin, uranyl acetate, and lead citrate were used for electron microscopy sample preparation. Coumarin 6 (Cou-6; 3-(2-benzothiazolyl)-7-(diethylamino)coumarin; 38215-36-0) was obtained from Merck (Darmstadt, Germany). Polypropylene ultracentrifuge tubes (Beckman Coulter) were used for extracellular vesicle isolation. Urea, dithiothreitol (DTT), iodoacetamide, sequencing-grade trypsin, and LC-MS grade acetonitrile and formic acid were purchased from Thermo Fisher Scientific. (1,1'-Dioctadecyl-3,3,3',3'-Tetramethylindotricarbocyanine Iodide) (DiR) (D4006) and (1,1'-Dioctadecyl-3,3,3',3'-Tetramethylindocarbocyanine Perchlorate (DiI) (D4010) fluorescent dyes were purchased from UElandy® and used for small extracellular vesicle (sEV) labeling and biodistribution analysis. Fluo-4 AM (Beyotime, S1061S) and 2′,7′-dichlorofluorescin diacetate (DCFH-DA; Sigma-Aldrich) were used for intracellular Ca²⁺ and reactive oxygen species detection, respectively. DAPI (Thermo Fisher Scientific), Alexa Fluor-conjugated phalloidin (488 or 568; Thermo Fisher Scientific), and mounting medium with DAPI (Thermo Fisher Scientific) were used for fluorescence imaging. Masson's Trichrome Stain Kit (G1340) for collagen fiber staining and hematoxylin and eosin (H&E) staining reagents were purchased from Solarbio.

Dulbecco’s Modified Eagle Medium (DMEM; Thermo Fisher Scientific), fetal bovine serum (FBS; Gibco), extracellular vesicle-depleted FBS (Thermo Fisher Scientific), and penicillin–streptomycin (Gibco) were used for cell culture. B16-F10 melanoma cells were maintained under standard culture conditions. Lipopolysaccharide (LPS; Sigma-Aldrich, L2880), interferon-γ (IFN-γ; Sigma-Aldrich), interleukin-4 (IL-4; Sino Biological), and interleukin-13 (IL-13; Sino Biological) were used for macrophage polarization. Calcein-AM/propidium iodide Live/Dead Cell Staining Kit (Sigma-Aldrich, 04511), Click-iT EdU Alexa Fluor Imaging Kit (Invitrogen, C10337/C10338), and Cell Counting Kit-8 (CCK-8; Dojindo, CK04) were used for cell viability and proliferation analysis. Recombinant mouse collagen I (USBiological, 154085) and recombinant GFP-tagged mouse integrin β1 (OriGene, MG227073) were obtained from the indicated suppliers. Cyclic RGD peptide, cRGDfK(C) (cRGD), and M2 macrophage-targeting peptide Cys-YEQDPWGVKWWY (M2pep) were synthesized by Sangon Biotech. Lipofectamine 3000 reagent (Thermo Fisher Scientific) was used for siRNA transfection. siRNAs targeting TRPV2, Rab11a, Rab11b, Rictor, and TLR4, together with miR-181a-5p inhibitors/mimics and corresponding control oligonucleotides, were synthesized by GenePharma or Shanghai DNA Bioscience Co. Ltd. unless otherwise specified. RNA extraction was performed using TRIzol reagent (Thermo Fisher Scientific), and reverse transcription was conducted using the RevertAid First Strand cDNA Synthesis Kit (Thermo Fisher Scientific). Quantitative PCR analysis was performed using SYBR Green Master Mix (Thermo Fisher Scientific).

For cytoskeletal, mechanotransduction, and signaling inhibition experiments, BAPTA-AM (GlpBio, GC13517), anti-integrin β1 blocking antibody (Sigma, MAB1997), CD44-blocking antibody (Thermo Fisher Scientific, MA4400), IL-10-neutralizing antibody (Proteintech, 69018-1-Ig), SB-431542 (GlpBio, GC11545), tranilast (TargetMol T2690, 53902-12-8), alpelisib (Selleckchem, S2814), JR-AB2-011 (Beyotime, SH3251), and galunisertib (Selleckchem, S2230) were prepared according to the manufacturers’ instructions. Stock solutions of small-molecule inhibitors were prepared in dimethyl sulfoxide, and the final dimethyl sulfoxide concentration did not exceed 0.1% (v/v). Corresponding vehicle-treated cells were included in all experiments.

Cytokine concentrations were determined using mouse TNF-α ELISA Kit (Sigma-Aldrich), mouse IL-6 ELISA Kit (Proteintech, KE10007), mouse IL-1β ELISA Kit (Invitrogen, BMS6002), mouse VEGF-A ELISA Kit (Invitrogen, BMS619-2), mouse TGF-β ELISA Kit (Invitrogen, BMS608-4), and mouse IL-10 ELISA Kit (Invitrogen, BMS614). For extracellular vesicle isolation and characterization, total protein concentrations were determined using the bicinchoninic acid (BCA) protein assay kit (Thermo Fisher Scientific). Size-exclusion chromatography was performed using Sepharose CL-4B resin (Sigma-Aldrich). Nanoparticle tracking analysis was performed using NanoSight instruments, and transmission electron microscopy (TEM) grids were prepared using carbon-coated copper grids.

RIPA lysis buffer, protease inhibitor cocktail, phosphatase inhibitor cocktail, sodium dodecyl sulfate polyacrylamide gel electrophoresis (SDS-PAGE) reagents, polyvinylidene fluoride (PVDF) membranes, and enhanced chemiluminescence (ECL) substrate (Thermo Fisher Scientific) were purchased from Thermo Fisher Scientific. Primary antibodies used for Western blotting, immunofluorescence staining, or immunohistochemical analysis included anti-integrin β1 (Sigma-Aldrich, MAB1997), anti-TRPV2 (Invitrogen, PA5-18989), anti-Rab11a (Invitrogen, MA5-49197), anti-CD63 (Invitrogen, PA5-92370), anti-CD9 (Invitrogen, MA5-31980), anti-CD81 (Invitrogen, MA5-32333), anti-Calnexin (Invitrogen, MA5-31501), anti-STAT3 (Invitrogen, MA1-13042), anti-FoxO1 (Invitrogen, MA5-14846), anti-TLR4 (Invitrogen, 48-2300), anti-NF-κB p65 (Proteintech, 66535-1-Ig), anti-phospho-NF-κB p65 (Invitrogen, 44-711G), anti-PI3K (HUABIO, JE04-11), anti-phospho-PI3K p85 (Tyr467)/p55 (Tyr199) (HUABIO, PSH01-38), anti-mTOR (Epizyme, M900010), anti-phospho-mTOR (Ser2481; Genuin Biotech., 62329), anti-CaMKII (Bioswamp, RMAB53545), anti-phospho-CaMKII (Thr286; HUABIO, JM11-07), anti-PYK2/PTK2B (HUABIO, SC06-15), anti-phospho-PYK2 (Tyr402; ZENBio, 310223), anti-iNOS (HUABIO, HA721583), anti-Arginase 1 (Arg-1; Proteintech, 6129-1-Ig), anti-IκB-α (Cell Signaling Technology, 4814), anti-Phospho-IκBα (Ser32) (Cell Signaling Technology, 2859), anti-CD68 (Bio-Rad, MCA1957), anti-CD163 (Invitrogen, 14-1631-82), anti-CD206 (Invitrogen, PA5-46994), anti-CD86 (eBioscience, 14-0862-82), anti-PD-L1 (Cell Signaling Technology, 13684), anti-CD8 (BioLegend, 100701), anti-CD31 (Invitrogen, 14-0311-82), anti-α-SMA (Invitrogen, MA5-11547), anti-GAPDH (Invitrogen, MA5-15738), and anti-β-tubulin (Invitrogen, MA5-16308) were used according to the manufacturers’ instructions.

Horseradish peroxidase (HRP)-conjugated secondary antibodies for Western blotting, including HRP-conjugated goat anti-mouse IgG (H+L; 31430), HRP-conjugated goat anti-rabbit IgG (H+L; 31460), and HRP-conjugated donkey anti-goat IgG (H+L; A15999), were purchased from Thermo Fisher Scientific. Fluorescence-conjugated secondary antibodies for immunofluorescence and immunohistochemistry, including Alexa Fluor 488- (A-11008, A-11001, A-21208), Alexa Fluor 555/568- (A-21428, A-21424), Alexa Fluor 594- (A-11012, A-11032), and Alexa Fluor 647- (A-21245, A-21235) conjugated goat anti-rabbit IgG, goat anti-mouse IgG, donkey anti-rabbit IgG, donkey anti-mouse IgG, and donkey anti-rat IgG, respectively, were purchased from Thermo Fisher Scientific.

For flow cytometry analysis, cells were first incubated with anti-CD16/32 antibody (clone 93) for Fc receptor blocking. Surface staining was performed using fluorophore-conjugated antibodies at 4 °C for 30 min. Antibodies included PE/Cy7-anti-CD45 (clone 30-F11), BV450-anti-CD11b (clone M1/70), PE-anti-F4/80 (clone Cl:A3-1), BV610-anti-Ly6G (clone 1A8), APC-anti-CD206 (clone C068C2), and AF488-anti-CD86 (clone GL1) purchased from eBioscience. Dead cells were excluded using 7-AAD staining solution (Thermo Fisher Scientific). All flow cytometry antibodies were purchased from Elabscience (Wuhan, China) unless otherwise indicated.

2. *Synthesis of methacrylated hyaluronic acid hydrogel*

Methacrylated hyaluronic acid (MeHA) with a substitution degree of approximately 30% was synthesized from sodium hyaluronate according to our previous report.^1^ Briefly, sodium hyaluronate was dissolved in deionized water and reacted with methacrylic anhydride under alkaline conditions while maintaining the pH at 8.0–8.5. After reaction, the solution was dialyzed against deionized water, lyophilized, and stored at −20 °C before use. The chemical structure and degree of methacrylation of MeHA were confirmed by ^1^H nuclear magnetic resonance spectroscopy (Agilent, DD2-500).

For hydrogel formation, 1 wt% MeHA prepolymer solution containing 0.05 wt% Irgacure 2959 (I2959, Ciba) was photocrosslinked under 365 nm UV light at 10 mW cm⁻^2^ for 15 min in confocal dishes, 24-well plates, thiolated glass substrates, or cylindrical molds (Corning Inc), depending on the experiment. The hydrogel was subsequently thiolated using mercaptosilatrane in 50% methanol for 2 h, followed by extensive washing with phosphate-buffered saline (PBS). Hydrogels were stored in sterile PBS at 4 °C before nanorod immobilization. For cRGD coupled-hydrogels of various mechanical stiffness, such as 2, 4 and 8 kPa, 1, 2 and 3 wt% MeHA prepolymers were pre-conjugated with the same concentrations of cRGD as mentioned below and the hydrogels were formed by the same photocrosslinking method.

3. *Synthesis of nanofiber-mimetic magnetic nanorods*

Nanofiber-mimetic nanorods (NMNs) were synthesized through sequential formation of FeOOH nanorods, silica coating, thermal reduction to silica-coated superparamagnetic iron oxide nanorods, and surface functionalization modified from a previous report.^2^ Briefly, FeCl3·6H₂O was dissolved in deionized water and heated at 87 °C without stirring for 24 h to generate FeOOH nanorods. The resulting brown precipitates were washed three times with Milli-Q water and dispersed in water.

FeOOH nanorods were then modified with polyacrylic acid (PAA) by overnight stirring at room temperature. After washing, PAA-modified FeOOH nanorods were coated with silica by reacting with TEOS in ethanol under magnetic stirring overnight. The FeOOH@SiO_2_ nanorods were washed with ethanol and water and subsequently reduced in DEG at 220 °C under nitrogen protection for 6 h to yield SPIONR@SiO_2_. The product was washed with ethanol and water and dispersed in ethanol. To introduce amine groups, SPIONR@SiO₂ was reacted with APTES under sonication for 2 h at room temperature. The resulting SPIONR@SiO_2_-NH_2_ was washed with ethanol and dispersed in ethanol. SPIONR@SiO_2_-NH_2_ was then reacted with Mal-NHS in PBS at pH 7.4 overnight to generate maleimide-functionalized SPIONR@SiO_2_-Mal. For cRGD conjugation, SPIONR@SiO_2_-Mal was incubated with 10 μg/mL of cRGD in PBS containing 0.5 mM TCEP overnight. Unreacted peptide was removed by repeated washing with PBS for zeta potential measurement by a dynamic light scattering instrument (Zetasizer, Malvern Panalytical).

4. *Coupling of NMNs to MeHA hydrogels and magnetic nanoalignment*

SPIONR@SiO_2_-Mal was added to thiolated MeHA hydrogels at different concentrations and reacted overnight under gentle shaking at room temperature. Immobilized SPIONR@SiO_2_ density was optimized by scanning electron microscope (SEM; Tescan MAIA3) analysis, and a coating density of 95 ± 8 particles µm^–2^ was selected to provide sufficient ligand presentation while preserving magnetic reorientation capacity. After SPIONR@SiO_2_ immobilization, the remaining maleimide groups were reacted with 10 μg/mL of cRGD in PBS containing TCEP overnight and the resulting immobilized NRs were referred to as nanofiber-mimetic nanorods (NMNs). The hydrogels were extensively washed with PBS before cell culture.

Hydrogels with randomly distributed NMNs were defined as nanoscale isotropic matrices (NIM). To generate nanoscale aligned matrices (NAM), hydrogels were exposed to a lateral magnetic field generated by permanent magnets. The magnetic field strength was measured using a handheld Gaussmeter (Model 410, Lake Shore Cryotronics) and maintained at approximately 40 mT unless otherwise stated. For SEM characterization, hydrogels were exposed to the magnetic field for 1 h before fixation and imaging. For cell culture experiments, magnetic stimulation was applied before seeding and maintained throughout the culture period to preserve nanoalignment.

5. *Preparation of collagen I-presenting matrices*

For the alternative ligand-presenting matrices, recombinant mouse collagen I was immobilized on maleimide-functionalized nanorods using the described Thiol-ene based conjugation procedure. Briefly, nanorods were incubated with collagen I at 25 μg mL⁻¹ in PBS overnight at 37 ^o^C, followed by repeated washing to remove unbound collagen. Matrices containing randomly distributed or magnetically aligned collagen I-presenting nanorods were designated Col1-NIM and Col1-NAM, respectively.

6. *Preparation of magnetically nonresponsive control matrices*

Magnetically nonresponsive control nanorods were prepared using FeOOH@SiO₂ nanorods without the diethylene glycol reduction step. The nanorods were functionalized with amine and maleimide groups, conjugated with cRGD, and immobilized on MeHA hydrogels at the same density as the magnetic NMNs. These matrices were cultured in the absence or presence of the same 40-mT magnetic field used for NAM generation.

7. *Physicochemical characterization of NMNs and hydrogels*

The morphology and size of FeOOH nanorods, SPIONR@SiO_2_, SPIONR@SiO_2_-NH_2_, SPIONR@SiO_2_-Mal, and NMNs were characterized by TEM (JEOL JEM-2010). High-angle annular dark-field scanning transmission electron microscopy (HAADF-STEM; Tecnai F20, FEI) equipped with energy-dispersive X-ray (EDX) spectroscopy was used for elemental mapping of Fe and Si. Hydrodynamic size and zeta potential were measured using dynamic light scattering (Malvern Instruments). Magnetic properties were analyzed by vibrating-sample magnetometry using a PPMS EverCool II-9T system. Crystal structure was examined by X-ray diffraction (XRD) using a Rigaku SmartLab diffractometer. Fourier-transform infrared (FTIR) spectroscopy was performed using a Nicolet iS50 FTIR spectrometer to confirm chemical conjugation.

Hydrogel surface morphology was observed by SEM after platinum sputtering. Angular distributions of NMNs were quantified using ImageJ from SEM images. Mechanical properties of hydrogels were measured using a rheometer (Malvern Kinexus) under small-amplitude oscillatory shear. The tests were performed using a 20 mm parallel-plate geometry at room temperature with a 100 µm gap size. After pre-equilibration for 15 min, the storage and loss moduli were measured at a strain of 1% and a frequency of 1 Hz, within the linear viscoelastic region established by a preliminary strain-sweep test. At least three independent hydrogels were measured per group.

8. *Equilibrium swelling ratio of NIM and NAM hydrogels*

NIM and NAM hydrogels were immersed in PBS (pH 7.4) at 37 °C for 48 h to reach swelling equilibrium. After gently removing excess surface liquid with filter paper, the hydrogels were immediately weighed to obtain the equilibrium wet mass (W_wet_). The samples were then lyophilized for 48 h and weighed again to obtain the dry mass (W_dry_). The equilibrium swelling ratio was calculated as:

Swelling ratio = $\frac{W_{wet}-W_{dry}}{W_{dry}}$

For pore-size analysis, swollen hydrogels were freeze-dried, sputter-coated with platinum, and imaged by SEM under identical acquisition conditions. Pore boundaries were segmented using ImageJ, and the equivalent pore diameter was quantified from five randomly selected fields per hydrogel. At least 30 pores were analyzed from each of four independently prepared hydrogels. The mean pore size from each hydrogel was treated as one biological replicate.

9. *Nanoindentation-based local modulus mapping*

Local mechanical properties of NIM and NAM hydrogels were measured in PBS using a Pavone nanoindentation system (Optics11 Life) equipped with a cantilever with a nominal spring constant of 0.41 N m⁻^1^ and a spherical-tip radius of 1 μm. Cantilever sensitivity and spring constant were calibrated before each experiment. Force–indentation curves were acquired at an approach rate of 10 μm s⁻^1^, a maximum loading force of 500 nN, and a maximum indentation depth of 10 µm. Young’s modulus was calculated by fitting the loading portion of each force curve using the Hertz contact model with a Poisson’s ratio of 0.45/0.49. For each hydrogel, measurements were collected from n = 6 hydrogels.

10. *Measurement of immobilized NMN density and cRGD density*

Immobilized NMN density on hydrogels was quantified from SEM images using ImageJ. Multiple randomly selected fields from at least three independent hydrogels were analyzed. cRGD conjugation density was quantified using Ellman’s reagent. Briefly, supernatants collected before and after peptide conjugation were mixed with 0.5 mg mL^–1^ DTNB and incubated for 15 min at room temperature in the dark. Absorbance was measured at 412 nm using a microplate reader (Thermo Fisher Scientific). Peptide consumption was calculated based on a standard curve, and cRGD density per nanorod was calculated using the immobilized NMN number.

11. *Integrin β1-based ligand-accessibility assay*

NIM and NAM hydrogels were incubated with recombinant GFP-tagged mouse integrin β1 at 10 μg mL⁻^1^ in PBS at pH 7.4 for 4 h at 37 ^o^C. Hydrogels were washed three times with PBS to remove unbound protein and imaged using confocal microscopy under identical laser power, gain, and exposure settings. Fluorescence-intensity line profiles and mean fluorescence intensities were quantified using ImageJ from five randomly selected fields from each of three independently prepared hydrogels. The mean fluorescence intensity from each hydrogel was treated as one biological replicate.

12. *AFM single-molecule force spectroscopy*

Atomic force microscopy was performed using a BioScope Catalyst AFM system operating in contact mode. Gold-coated OBL AFM tips with a nominal spring constant of 30 mN m^–1^ were functionalized with recombinant mouse integrin β1 by incubation in integrin β1 solution at 1 mg mL^–1^ for 4–6 h at room temperature. Tips were gently rinsed with Milli-Q water before use. Force–distance curves were acquired on NIM and NAM hydrogels in aqueous conditions at a retraction velocity of 1 µm s^–1^. Maximum rupture forces were extracted from retraction curves using Nanoscope Analysis software. At least 40 force curves from three independent hydrogels were analyzed per group.

13. *TEM analysis of melanoma ECM ultrastructure*

All animal procedures were approved by the Scientific Ethics Committee of the Academic Committee of Ocean University of China (OUC-AE-2024-091 and OUC-AE-2026-205). To examine native ECM nanoarchitecture, B16-F10 syngeneic melanoma tumors were harvested on day 10, when the tumor volumes reached approximately 1000 mm^3^. Intratumoral and peri-tumoral regions from three mice were dissected into approximately 1 mm^3^ tissue blocks and immediately fixed in 2.5% glutaraldehyde in phosphate buffer at 4 °C overnight. Samples were washed with phosphate buffer and post-fixed with 1% osmium tetroxide for 1–2 h. Tissues were dehydrated through a graded ethanol series, treated with propylene oxide, embedded in epoxy resin, and polymerized at 55–60 °C. Ultrathin sections of approximately 70 nm were prepared using an ultramicrotome (Leica Microsystems), mounted on copper grids (Electron Microscopy Science), stained with uranyl acetate and lead citrate, and imaged by TEM. ECM fiber diameter and alignment were quantified using ImageJ. For each tumor, five randomly selected TEM fields from each region were examined, and a total of 20 ECM fibers were quantified for fiber diameter and orientation. Individual animals were treated as biological replicates, whereas multiple fields and fibers were analyzed as subsamples within each animal.

14. *Cell culture and TAM isolation*

The B16-F10 mouse melanoma cell line was obtained from the American Type Culture Collection and cultured in DMEM supplemented with 10% heat-inactivated FBS and 1% penicillin–streptomycin at 37 °C in a humidified incubator with 5% CO_2_. TAMs were isolated from B16-F10 syngeneic melanoma tumors by fluorescence-activated cell sorting. Tumors were minced and enzymatically digested using collagenase IV (Worthington, 1 mg/mL), DNase I (Roche, 0.2 mg/mL), and hyaluronidase (Tokyo Chemical Industry) in serum-free medium at 37 °C with gentle agitation. Cell suspensions were filtered through 70 µm cell strainers (Corning Inc.), red blood cells were lysed by Red Blood Cell Lysis Buffer (Biolegend), and single-cell suspensions were stained for flow cytometric sorting. TAMs were isolated as viable CD45⁺CD11b⁺Ly6G⁻F4/80⁺ cells. CD86 and CD206 were subsequently analyzed for phenotypic characterization and were not used as sorting criteria. The following fluorescently labeled monoclonal antibodies were purchased from Elabscience (Wuhan, China): PE-Cy7 anti-mouse CD45 (clone 30-F11, cat. no. E-AB-F1136H); PE anti-mouse F4/80 (clone CI:A3-1, cat. no. E-AB-F0995UD); PE anti-mouse CD86 (clone GL-1, cat. no. E-AB-F0994D); APC anti-human CD206/MMR (clone 15-2, cat. no. E-AB-F1161E); APC anti-mouse Ly6G (clone 1A8, cat. no. E-AB-F1108UE); and Elab Fluor® Violet 450 anti-mouse/human/monkey CD11b (clone M1/70, cat. no. E-AB-F1081Q). Sorted cells were cultured in DMEM supplemented with 10% FBS, 1% PS, and recombinant mouse CSF-1 to maintain macrophage survival. Cell viability was assessed using the CCK-8 assay (Sparkjade).

15. *Live/dead staining assay*

Cells cultured on NIM and NAM hydrogels were stained using a Calcein-AM/PI Live/Dead Cell Double Staining Kit according to the manufacturer’s instructions. Briefly, cells were incubated with staining solution containing Calcein-AM (2 μM) and PI (4 μM) for 20–30 min at 37 °C in the dark. After washing with PBS, fluorescence images were acquired using a fluorescence microscope. Live cells (green fluorescence) and dead cells (red fluorescence) were quantified using ImageJ, and cell viability was calculated as the percentage of Calcein-AM-positive cells among total cells.

16. *Matrix priming and macrophage polarization*

TAMs were seeded on NIM or NAM hydrogels at approximately 10,000 cells cm⁻^2^. Cell adhesion and spreading were assessed after 24 h. For polarization experiments, TAMs were first cultured on hydrogels for 12 h in basal medium to allow attachment and then cultured for an additional 24 h under pro-inflammatory or anti-inflammatory conditions. Pro-inflammatory stimulation was induced using lipopolysaccharide (LPS) and recombinant interferon (IFN)-γ. Anti-inflammatory stimulation was induced using recombinant IL-4 and IL-13. After treatment, cells were fixed for immunofluorescence staining or collected for qRT-PCR and immunoblotting. Culture supernatants were collected for cytokine analysis or sEV isolation as indicated. For dynamic switching experiments, TAMs were cultured on NIM or NAM for the first 12 h and then switched to the opposite nanoalignment state for an additional 24 h. NIM-to-NAM switching was achieved by applying the lateral magnetic field, whereas NAM-to-NIM switching was achieved by removing the magnetic field and allowing nanorods to return to a less aligned state. Cells were then analyzed for macrophage polarization markers by immunofluorescence, qRT-PCR, and cytokine secretion assays.

17. *Antibody blocking and pharmacological inhibition*

TAMs were pretreated with an integrin β1-blocking antibody, CD44-blocking antibody, or corresponding isotype IgG at 5 μg mL⁻¹ for 1 h at 37 °C before seeding on the hydrogels. The antibodies were maintained throughout the subsequent 24-h matrix-priming period. Tranilast, alpelisib, and JR-AB2-011 were used at 20 μM, 2.5 nM, and 10 μM, respectively. BAPTA-AM was used at 10 μM. Unless otherwise stated, inhibitors were added during matrix priming and maintained during the 24-h conditioned-medium collection period. Vehicle-treated cells received an equivalent volume of dimethyl sulfoxide. For cytokine-pathway blockade in recipient macrophages, cells were pretreated with IL-10-neutralizing antibody at 1:50 dilution of the stock solution or SB-431542 at 10 μM for 24 h before sEV treatment. The antibody or inhibitor was maintained during the subsequent 36-h sEV-treatment period.

18. *siRNA and miRNA inhibitor transfection*

Trpv2, Rab11a, Rab11b, Tlr4, Rictor and miR-181a-5p knockdown experiments were performed using Lipofectamine 3000 or an equivalent transfection reagent according to the manufacturer’s protocol. All relevant sequences are listed in Table S1. TAMs were seeded in antibiotic-free medium before transfection. siRNA or miRNA inhibitor was added at the optimized final concentration, and cells were incubated for 24–48 h before downstream experiments. Rab11a- or Rab11b-silenced TAMs were seeded onto NIM or NAM hydrogels for analysis of CD63 distribution and sEV release. For the generation of miR-181a-5p-depleted sEV^NAM^, donor TAMs were transfected with miR-181a-5p inhibitor or negative control inhibitor before NAM priming and sEV collection. Knockdown efficiency was verified by qRT-PCR and/or Western blotting as appropriate. Rab11a knockdown was confirmed at the protein level. The functional effect of Rictor-targeting siRNA on mTORC2-associated signaling was assessed by phosphorylated mTOR Ser2481 relative to total mTOR.

19. *Immunofluorescence staining*

Cells or tissue sections were fixed with 4% paraformaldehyde, washed with PBS, permeabilized with 0.25% Triton X-100, and blocked with 1% bovine serum albumin. Samples were incubated with primary antibodies overnight at 4 °C, followed by fluorophore-conjugated secondary antibodies for 1–2 h at room temperature. F-actin was stained with phalloidin, and nuclei were stained with DAPI. Primary antibodies used for cell staining included anti-integrin β1, anti-FAK, anti-iNOS, anti-Arg-1, anti-Rab11a, anti-CD63, anti-STAT3, anti-FoxO1, anti-TLR4, and anti-phospho-NF-κB p65. Tissue sections were stained with antibodies against CD68, CD163, CD86, CD206, PD-L1, CD8, α-SMA, CD31, CD9, and CD63 as indicated. Images were acquired using confocal microscopy.

20. *Calcium influx assay and imaging*

Calcium influx was measured using Fluo-4 AM. TAMs cultured on NIM or NAM hydrogels with or without tranilast treatment were incubated with Fluo-4 AM working solution in serum-free medium at 37 °C in the dark. After dye loading, cells were washed and incubated in fresh imaging buffer to allow de-esterification. Time-lapse fluorescence imaging was performed using confocal microscopy (Nikon Eclipse Ti2) with excitation at 488 nm. Fluorescence intensity was recorded over time and normalized to the initial fluorescence intensity of each cell. Quantification was performed using ImageJ. At least 20 cells from three independent hydrogels were analyzed per group.

21. *Western blotting*

Cells or isolated sEVs were lysed in RIPA buffer containing protease and phosphatase inhibitors. Protein concentration was determined by BCA assay. Equal amounts of protein were separated by SDS–PAGE, transferred to PVDF membranes, and blocked with 5% non-fat milk or bovine serum albumin. Membranes were incubated with primary antibodies overnight at 4 °C and HRP-conjugated secondary antibodies at room temperature. Signals were detected using enhanced chemiluminescence. Antibodies against GAPDH, β-actin, β-tubulin, CD9, CD63, CD81, TRPV2, Rab11a, PI3K, phosphorylated PI3K, mTOR, phosphorylated mTOR Ser2481, CaMKII, phosphorylated CaMKII Thr286, PYK2/PTK2B, phosphorylated PYK2 Tyr402, STAT3, phosphorylated STAT3, TLR4, NF-κB p65, phosphorylated NF-κB p65, IκB-α, and phosphorylated IκB-α were used as indicated. Band intensities were quantified using ImageJ. Phosphorylated proteins were normalized to their corresponding total proteins, whereas total cellular proteins were normalized to the indicated loading controls. Each biological replicate represented an independently prepared cell lysate or sEV preparation.

22. *GTP-bound Rab11a pull-down assay*

Active GTP-bound Rab11a was isolated using a Rab11 activation pull-down assay according to the manufacturer’s instructions. TAM lysates were prepared under conditions preserving the GTP-bound state and incubated with the corresponding Rab-binding capture reagent at 4 °C. Bound proteins were washed, eluted, and detected by Western blotting using an anti-Rab11a antibody. GTP-Rab11a intensity was normalized to total Rab11a in the corresponding input lysate. Total Rab11a and RICTOR were normalized to β-tubulin, whereas mTORC2-associated activation was quantified as phosphorylated mTOR Ser2481 relative to total mTOR. Ratios were expressed relative to the NAM + siRNA Negative Controls (si-NC) group.

23. *qRT-PCR and RNA sequencing*

qRT-PCR was performed according to a previous report.^3^ Basically, TRIzol reagent (Invitrogen) was used to extract all samples’ RNA according to the manufacturer's instructions. The concentrations of extracted RNA were measured by a Nanodrop One spectrophotometer (Nanodrop Technologies). Then, the extracted RNA was reversed transcribed into cDNA using a RevertAid First Strand cDNA Synthesis Kit according to the manufacturer’s instructions (Thermo Fisher Scientific). After that, we carried out RT-qPCR using Taqman primers probing GAPDH and specific genes including TRPV1–6, NOS2, CD80, ARG1, YM2, TLR4, RAB11A, RAB11B. (Applied Biosystems 7300 Real-Time PCR system, Thermo Fisher scientific) Sequences of primers and probes used are listed in Table S1. Gene expression levels were first normalized to those of GAPDH and then normalized to the expression level of control group at the same time point to Ct values, respectively. ΔCt was calculated as Ct_target_−Ct_GAPDH_, and relative expression was calculated as 2^−ΔCt^. Ratio of expression was determined as the ratio of relative expression of the target gene in the treated group to the control group.

For whole-transcriptome sequencing, the NIM- or NAM-primed TAMs were sent to the Beijing Genomics Institute (BGI) Group for cDNA preparation and whole-transcriptome sequencing. Three independent biological samples were analyzed per group. Library construction and sequencing were performed according to BGI’s standardized quality-control procedures. Double-stranded cDNA libraries that met the quality requirements were sequenced on the DNBSEQ platform, generating at least 20 million clean reads per sample. Reads were mapped to the mouse mm10 reference genome. Differential-expression analysis was performed using DESeq2 based on gene-level read counts from independent biological replicates. The resulting (p) values were adjusted for multiple testing using the Benjamini-Hochberg false-discovery-rate method. Genes with a Q value < 0.05 and an absolute (log2)-fold change of ≥ 1 were considered differentially expressed. Gene Ontology (GO) and Kyoto Encyclopedia of Genes and Genomes (KEGG) enrichment analyses were performed using the differentially expressed genes, whereas gene set enrichment analysis (GSEA) was conducted using ranked gene-expression data. GO terms, KEGG pathways, and gene sets with a Q value ≤ 0.05 were considered significantly enriched.

24. *Isolation of sEVs from TAM-conditioned media*

TAMs were cultured in medium containing EV-depleted fetal bovine serum. Conditioned medium was collected after 24 h and centrifuged sequentially at 300 × *g* to remove cells, 2,000 × *g* to remove dead cells and debris, and 10,000 × *g* to remove larger vesicles and apoptotic bodies. The resulting supernatant was filtered through a 0.22-μm membrane and ultracentrifuged at 120,000 × *g* for 2 h at 4 °C. The sEV pellet was washed with PBS and ultracentrifuged again under the same conditions. Purified sEVs were resuspended in PBS and stored at −80 °C for short-term use. The number of viable adherent donor cells was determined immediately before conditioned-medium collection using a trypan blue (0.4%; Gibco™) exclusion test of cell viability. sEV particle output was normalized to the corresponding viable adherent donor-cell number. In accordance with MISEV2023, the isolated particles are referred to as sEVs because their endosomal origin was not directly established. Donor-cell culture conditions, isolation, normalization, storage, and functional dosing were reported, and sEVs were characterized by NTA, TEM, and immunoblotting for EV-associated proteins. The current SI reports differential ultracentrifugation, viable-cell normalization, NTA, TEM, and EV-marker characterization as the core sEV workflow.

25. *Purification of sEVs by Size Exclusion Chromatography (SEC)*

SEC was performed only for validation of sEV purity and was not used for subsequent functional experiments. The procedure followed a previous report.^4^ Sepharose CL-4B resin (Solarbio) was suspended in phosphate-buffered saline (PBS; 10 mM phosphate and 150 mM NaCl, pH 7.2), thoroughly degassed, and packed under gravity into disposable syringes fitted with a 30 µm bottom frit. Columns were prepared with a bed volume of 10 mL and equilibrated with at least two bed volumes of 0.22 µm-filtered PBS before sample loading. 500 µL extracellular samples were gently loaded onto the top of the column without disturbing the resin bed. After the sample had completely entered the column, PBS was added and the sample was eluted under gravity flow. Sequential fractions of 0.5 mL were collected and maintained at 4 °C until further analysis. Individual fractions were analyzed for particle concentration and size by NTA, EV-associated markers such as CD63 and CD81 by western blotting, and total protein content by BCA assay.

26. *Nanoparticle tracking analysis (NTA) and TEM characterization of sEVs*

Purified sEVs were diluted in particle-free PBS and analyzed using a NanoSight NS300 instrument. Particle concentration and size distribution were calculated using NanoSight NTA software. Three independently prepared conditioned-medium and sEV samples were analyzed per group (n=3 biological replicates). For each biological replicate, three 60s videos were recorded and averaged to obtain a single particle concentration and size-distribution value. For TEM imaging, sEV suspensions were placed on carbon-coated copper grids, negatively stained with uranyl acetate or phosphotungstic acid, dried, and imaged by TEM. For quantitative comparison, sEV number was normalized to donor cell number or plasma volume, depending on the experiment.

27. *BioTEM sample preparation for visualization of sEV release*

TAMs cultured on NIM or NAM hydrogels were collected and fixed in 2.5% glutaraldehyde in phosphate buffer. Cell pellets were post-fixed with 1% osmium tetroxide, dehydrated through a graded ethanol series, treated with propylene oxide, embedded in Epon resin, and polymerized. Ultrathin sections were prepared on copper grids and stained with platinum blue and lead citrate before TEM observation. Vesicle-like structures near the plasma membrane were imaged and quantified from multiple cells per group.

28. *Recipient macrophage treatment with TAM-derived sEVs*

Recipient macrophages were seeded in EV-depleted medium and treated with sEV^NIM^, sEV^NAM^, sEV^NAM^ generated in the presence of JR-AB2-011, or miR-181a-5p-depleted sEV^NAM^. sEV dose was normalized by particle number as determined by NTA or by protein amount, where indicated. For polarization assays, recipient macrophages were treated with sEVs in the presence of pro-inflammatory stimulation or anti-inflammatory stimulation for 36 h. Cells were then analyzed by immunofluorescence staining, qRT-PCR, Western blotting, ROS assay, and cytokine assays. Control cells were treated with PBS or vehicle in EV-depleted medium. For CCK-8 assay, cells were seeded into 96-well plates at a density of 5 × 10³ cells per well and treated with the indicated formulations. After incubation, 10 μL of CCK-8 solution was added to each well and incubated for 1–2 h at 37 °C according to the manufacturer’s protocol. Absorbance at 450 nm was measured using a microplate reader. Cell viability was calculated relative to untreated control cells. Three independent biological replicates were performed for each condition

29. *Prediction of miR-181a-5p binding sites in TLR4 3′UTR*

Potential binding sites between miR-181a-5p and the mouse *Tlr4* 3′ untranslated region were predicted using TargetScan and miRanda. The mouse TLR4 3′UTR sequence was used as the input sequence. Predicted binding sites were selected based on seed sequence complementarity and conservation scores. The predicted binding alignment was visualized for presentation. Functional involvement of the predicted miR-181a-5p–TLR4 interaction was assessed by miR-181a-5p knockdown in donor TAMs, sEV treatment of recipient macrophages, and comparison with TLR4 siRNA knockdown in recipient macrophages.

30. *ROS measurement*

Intracellular reactive oxygen species were measured using DCFH-DA. After treatment, cells were incubated with DCFH-DA working solution at 37 °C in the dark, washed with PBS, and imaged by fluorescence microscopy or analyzed by flow cytometry. For image-based quantification, integrated optical density was measured using ImageJ or Image-Pro Plus and normalized to cell area.

31. *ELISA analysis of cytokines*

Cytokine concentrations in conditioned media or isolated sEV lysates were measured using ELISA kits according to the manufacturer’s instructions. TNF-α, IL-6, IL-1β, VEGF-A, TGF-β, and IL-10 were analyzed as indicated. For sEV cytokine analysis, equal numbers of NTA-quantified sEV particles were lysed for each group before ELISA measurement, thereby enabling particle-normalized comparison of cytokine abundance.

32. *Small RNA sequencing and miRNA analysis of sEVs*

For miRNA profiling, sEVs were isolated from NIM-primed TAMs, NAM-primed TAMs, and NAM-primed TAMs treated with JR-AB2-011. To distinguish changes in sEV cargo composition from differences in total sEV production, equal numbers of sEV particles, as determined by NTA, were used for RNA extraction in all groups. Thus, differences in miRNA abundance represent particle-normalized changes in the recovered sEV population rather than differences caused solely by total vesicle yield. Total sEV RNA, including small RNA, was extracted using a commercial small RNA isolation kit according to the manufacturer’s protocol. siRNA sequences were listed in Table S1. RNA quantity and quality were assessed using a Bioanalyzer small RNA chip. Small RNA libraries were prepared using a commercial small RNA library preparation kit and sequenced on a DNBSEQ platform. Reads were trimmed, filtered, and mapped to the mouse genome and miRBase database. miRNA expression was normalized as TPM. Differentially expressed miRNAs were identified using a false discovery rate-adjusted Q value < 0.05. miRNA-associated pathway enrichment analysis and GSEA were performed using predicted or validated target genes. For sEV miRNA analysis, equal numbers of NTA-quantified sEV particles were used, and a spike-in small RNA control was used consistently for normalization. qPCR was performed using miR-181a-5p-specific primers.

33. *LC–MS/MS proteomic analysis*

Sequencing-grade modified trypsin (Promega) was used for protein digestion before LC-MS/MS analysis. Proteins extracted from purified sEVs were quantified by BCA assay, and 50 μg of protein per sample was denatured in 8 M urea, reduced with DTT and alkylated with iodoacetamide. Proteomic comparisons were performed using equal total protein input and describe relative differences in protein-cargo composition rather than per-particle protein abundance. DTT was added again and the proteins were captured on magnetic beads and digested overnight with trypsin at 37 °C. The resulting peptides were recovered, desalted using C18 columns, vacuum-dried, and reconstituted in 0.1% formic acid. Peptides were separated using a Vanquish Neo UHPLC system equipped with a C18 trap column and PepMap analytical column and analyzed on an Orbitrap Astral Zoom mass spectrometer in data-independent acquisition mode. Full MS scans were acquired over *m/z* 380–980 at a resolution of 240,000, followed by Astral MS/MS acquisition over *m/z* 150–2,000 using 300 isolation windows of 2 *m/z* and a normalized collision energy of 27%. Raw data were processed using DIA-NN v2.3.2 with trypsin specificity and one allowed missed cleavage. Carbamidomethylation of cysteine was set as a fixed modification, whereas methionine oxidation and protein N-terminal acetylation were treated as variable modifications. Peptide and protein identifications were controlled at a 1% false-discovery rate. Differential protein abundance, principal component analysis, heatmap visualization, and Gene Ontology-based gene-set enrichment analysis were subsequently performed using three independent biological replicates per group.

34. *Preparation of M2-like TAM-targeting inhibitor- and siRNA-loaded lipid nanocarriers*

Tranilast- or galunisertib-loaded lipid nanocarriers were prepared using the thin-film hydration and membrane-extrusion method. Hydrogenated soybean phosphatidylcholine (HSPC; MedChemExpress), 1,2-distearoyl-sn-glycero-3-phosphoglycerol (DSPG; MedChemExpress), and cholesterol (Sigma) were mixed at a molar ratio of 55:10:35 and dissolved in a chloroform mixture, according to a previous report.^5^ Tranilast or galunisertib was added to the lipid-containing organic phase at a drug-to-lipid mass ratio of 1:10. The organic solvent was removed by rotary evaporation to form a uniform lipid film, followed by vacuum drying. The lipid film was hydrated with PBS at 60 ^o^C. The resulting suspension was sequentially extruded through polycarbonate membranes with pore sizes of 400, 200, and 100 nm to obtain lipid nanocarriers with a uniform size distribution. For M2-like TAM targeting, M2pep-conjugated 1,2-Distearoyl-sn-glycero-3-phosphorylethanolamine (DSPE)–PEG2000 was incorporated into the preformed lipid nanocarriers by incubating at 60 ^o^C for 30 min. Cou-6-loaded lipid nanocarriers were prepared following the same methods as aforementioned, except that Cou-6 was added before formation of the thin film. Unencapsulated drug and free peptide–lipid conjugate were removed by dialysis.

Rab11a-siRNA-loaded lipid nanocarriers were prepared separately using the pH-responsive charge-reversible lipid dioleoylglycerophosphate–diethylenediamine conjugate (DOP-DEDA; MedChemExpress), according to a previously reported method.^6^ DOP-DEDA, 1,2-dipalmitoyl-sn-glycero-3-phosphocholine (DPPC), and cholesterol were dissolved in tert-butanol at a molar ratio of 45:10:45 and a final lipid concentration of 10 mM. Rab11a-siRNA was dissolved separately in 1 mM sodium citrate (pH 4.0). The lipid and siRNA solutions were separately preheated to 40 °C and then rapidly combined at a volume ratio of 1:5 and immediately vortexed for 30 s to induce lipid nanocarrier self-assembly. The resulting lipid nanocarriers were incubated at room temperature for 15 min and subsequently dialyzed or buffer-exchanged into RNase-free PBS at pH 7.4 to remove tert-butanol and unencapsulated siRNA. The purified DSPE–PEG2000–M2pep conjugate was added to the preformed, buffer-exchanged Rab11a-siRNA-loaded lipid nanocarriers at 0.5 mol% relative to the total lipid content. The mixture was incubated at 37 °C for 60 min with gentle agitation to facilitate insertion of the DSPE lipid anchor into the outer lipid layer of the lipid nanocarriers. Following purification, the formulations were characterized by hydrodynamic diameter, polydispersity index, zeta potential, and siRNA encapsulation efficiency.

35. *Subcutaneous implantation of hydrogels and skin-response model*

8 to 10-week-old BALB/c mice were anesthetized with a mixture of 2.0%–2.5% isoflurane (RWD Life Science) in oxygen, and a ~1.5 cm dorsal skin incision was made to create a subcutaneous pocket. Sterile NIM or NAM hydrogels were implanted into the pocket, and the incision was closed. After 24 h, the implants and surrounding tissues were harvested for immunofluorescence staining and RT-qPCR analysis. The recruitment, adhesion, and polarization states of infiltrating macrophages were evaluated by staining for macrophage and polarization-associated markers.

36. *In vivo melanoma model and sEV administration*

6 to 7-week-old C57BL/6J mice were housed under specific pathogen-free conditions at 22~24 °C and 50~60 % with a 12 h light/12 h dark cycle and free access to food and water. Mice were randomly assigned to the experimental groups before treatment. B16-F10 syngeneic melanoma tumors were generated by subcutaneous injection of B16-F10 cells into mice. Tumor size was measured using a digital caliper, and tumor volume was calculated using the standard formula (length × width^2^/2). Mice were monitored regularly for body weight, general appearance, mobility, and signs of pain or distress. Humane endpoints were defined according to the institutionally approved criteria, including a maximum tumor volume exceeding 1500 mm^3^. For sEV administration, purified sEVs from NIM- or NAM-primed TAMs were resuspended in sterile PBS (20 μg sEVs in 100 μL PBS) and intravenously injected through the tail vein every 3 days. Control mice received PBS. At the endpoint, mice were deeply anesthetized with inhalation of 2.0%–2.5% isoflurane in oxygen and subsequently euthanized by cervical dislocation. After that, tumors were excised, weighed, fixed, and processed for histology and immunostaining. Survival was monitored according to approved humane endpoints.

For the subcutaneous skin assay, PBS, sEV^NIM^, sEV^NAM^, or miR-181a-5p-depleted sEV^NAM^ was injected subcutaneously into 6- to 7-week-old BALB/c mice every 3 days for 14 days. Skin surrounding the injection site was harvested for immunofluorescence staining, hematoxylin and eosin staining, Masson’s trichrome staining, and dermal thickness analysis. Each mouse was considered one biological replicate, while serial tumor measurements and multiple tissue sections or fields from the same mouse were treated as repeated observations or subsamples.

37. *EdU incorporation assay*

To evaluate tumor cell proliferation, mice received an intraperitoneal injection of EdU (50 mg kg⁻¹) 2 h before sacrifice. Tumor tissues were collected, fixed with 4% paraformaldehyde, embedded, and sectioned. EdU incorporation was detected using a Click-iT EdU Imaging Kit according to the manufacturer’s protocol. Nuclei were counterstained with DAPI. The percentage of proliferating cells was quantified as EdU-positive nuclei divided by total DAPI-positive nuclei from randomly selected tumor regions.

38. *In vivo administration of TAM-targeting lipid nanocarriers*

When tumors reached the indicated size, melanoma-bearing mice were randomly assigned to receive empty M2pep-modified lipid nanocarriers loaded with tranilast, Rab11a-siRNA, or galunisertib by tail-vein injection. Treatments were administered twice per week for 2 weeks. Drug and siRNA doses were calculated based on the encapsulated cargo. Tumor growth was monitored throughout treatment. At the endpoint, tumors and blood samples were collected for analysis of sEV abundance, ECM deposition, TAM polarization, PD-L1 expression, CD8⁺ T-cell infiltration, and tumor burden. Flow-cytometric assessment of the uptaken Cou-6-labeled M2pep-modified lipid nanocarriers was intravenously administered to melanoma-bearing mice at a lipid nanocarrier dose of 1.67mg/mL. Tumors were collected at 24 h after injection, mechanically and enzymatically dissociated, filtered through 70 μm strainers, and stained with a viability dye and antibodies against CD45, CD11b, F4/80, CD206, and CD86. After exclusion of debris, doublets, and dead cells, CD206⁺ TAMs were identified as CD45⁺CD11b⁺F4/80⁺ Ly6G^-^CD206⁺. CD86⁺ myeloid cells were identified as CD206⁺CD11b⁺ly6G^-^, and CD45⁻FSCʰ^igh^cells were classified as tumor-cell-enriched rather than definitively pure tumor cells. Cou-6 positivity was quantified within each subset and relative to the total viable tumor-derived population. Unlabeled lipid nanocarriers and fluorescence-minus-one controls were used to define positive gates.

39. *Isolation and characterization of circulating plasma sEVs*

Blood was collected from treated melanoma-bearing mice into EDTA-coated tubes. Samples were centrifuged at low speed to remove blood cells, followed by additional centrifugation to remove platelets and cellular debris. Plasma was diluted with PBS, filtered through a 0.22 µm filter when appropriate, and subjected to ultracentrifugation to isolate circulating sEVs. For ultracentrifugation-based isolation, plasma supernatants were centrifuged at 120,000 × g for 2 h at 4 °C, washed with PBS, and centrifuged again under the same conditions. The final pellet was resuspended in PBS. Particle concentration and size distribution were measured by NTA and normalized to plasma volume. Where indicated, circulating sEV markers CD9 and CD63 were analyzed by Western blotting or immunostaining.

40. *In vivo biodistribution of DiR-labeled sEVs*

Purified sEVs were labeled with DiR for in vivo imaging or DiI for tissue-section analysis. For DiI labeling, approximately 50 μg sEV protein were incubated with 20 μM DiI at 37 °C for 30 min in the dark. DiR labeling was performed similarly using the indicated dye concentration. Free dye was removed by ultracentrifugation and labeled sEVs were resuspended in sterile PBS. Melanoma-bearing mice received labeled sEVs by tail-vein injection. DiR fluorescence was monitored by a small animal in vivo imaging system (Aniview SE, Guangzhou Boluteng Biotechnology Co., Ltd.) at 0, 2, 6, 24, and 48 h. At the indicated endpoints, organs and tumors were collected for ex vivo fluorescence imaging. Fluorescence intensity was quantified using identical regions of interest and imaging settings. For tissue colocalization, tumors were collected after administration of DiI-labeled sEVs, fixed, cryosectioned, and immunostained for CD68 and CD163. Confocal images were acquired using identical settings, and colocalization between DiI-positive sEV signals and CD68⁺CD163⁺ TAMs were quantified using ImageJ. Dye-only samples subjected to the same purification procedure were used as negative controls to exclude fluorescence from residual free dye.

41. *Histology and immunohistochemistry*

Tumors, skin tissues, and major organs were fixed in 4% paraformaldehyde, dehydrated through graded ethanol, cleared in xylene, and embedded in paraffin. Sections were deparaffinized, rehydrated, and subjected to hematoxylin and eosin staining or Masson’s trichrome staining. For immunostaining, antigen retrieval was performed in citrate buffer. Endogenous peroxidase activity was quenched when required. Sections were blocked and incubated with primary antibodies overnight at 4 °C, followed by appropriate secondary antibodies. For immunofluorescence staining, nuclei were counterstained with DAPI. Images were acquired using fluorescence microscopy or confocal microscopy.

42. *Image quantification and colocalization analysis*

Image analysis was performed using ImageJ. Cell spreading area was quantified from F-actin-stained images by manually or semi-automatically segmenting cell boundaries. Shape factor was calculated based on the cell length-to-width ratio, or another stated geometric descriptor and kept consistent across groups. Fiber or cell orientation was quantified using OrientationJ or equivalent ImageJ plugins. Rab11a/CD63 colocalization was quantified using Manders’ overlap coefficient with thresholding applied consistently across samples. Nuclear localization of STAT3 and FoxO1 was quantified by calculating nuclear-to-cytoplasmic fluorescence intensity ratios using DAPI-defined nuclear masks. Perinuclear CD63 accumulation was quantified by defining a perinuclear region surrounding the DAPI-positive nucleus and calculating the percentage of total CD63 signal within that region. The numbers of cells, fields, and independent hydrogels analyzed are specified in the corresponding figure legends. Cells and fields obtained from the same hydrogel were treated as subsamples, and the mean value from each independently prepared hydrogel was treated as one biological replicate. Tumor immunofluorescence images were quantified from randomly selected fields within tumor boundaries. CD8⁺ T-cell infiltration, PD-L1-positive area, TAM abundance, M1/M2 ratio, collagen deposition, and CD9/CD63 staining intensity were quantified using identical thresholds across groups. For tissue-image quantification, three nonconsecutive sections per animal and five randomly selected fields per section were analyzed unless otherwise indicated. Measurements from the same animal were averaged to generate one biological replicate. The number of animals analyzed in each experiment is specified in the corresponding figure legend. Measurements from the same animal were averaged to generate one value for statistical analysis.

43. *Statistical analysis*

All quantitative data are presented as mean ± standard deviation unless otherwise indicated. Statistical analyses were performed using GraphPad Prism. For comparisons between two groups, unpaired two-tailed Student’s t tests were used. For multiple-group comparisons, one-way or two-way ANOVA followed by appropriate post hoc tests was performed. Kaplan-Meier survival curves were analyzed using the log-rank test. Sample sizes and statistical tests are indicated in the figure legends. A p-value ≤ 0.05 was considered statistically significant.

**Table S1 Primer and RNA-interference oligonucleotide sequences used in this study.**

| Target gene | Primer | Sequences (5’🡪3’) |
| --- | --- | --- |
| *Trpv1* | F | CAAACTCCACCCCACACTGA |
|  | R | AGGCCAAGACCCCAATCTTC |
| *Trpv2* | F | GTTTGACCGTGACCGACTCT |
|  | R | GAGCCTTCTGTGTATGCCGA |
| *Trpv4* | F | ACTGGAACCAGAACTTGGGC |
|  | R | AGGACCAACGATCCCTACGAA |
| *Trpv6* | F | GGGGTTAATACTCTGCCTATGG |
|  | R | GCACCTCACATCCTTCAAACTT |
| *Nos2* | F | GAGACAGGGAAGTCTGAAGCAC |
|  | R | CCAGCAGTAGTTGCTCCTCTTC |
| *Cd80* | F | CCTCAAGTTTCCATGTCCAAGGC |
|  | R | GAGGAGAGTTGTAACGGCAAGG |
| *Arg-1* | F | CATTGGCTTGCGAGACGTAGAC |
|  | R | GCTGAAGGTCTCTTCCATCACC |
| *Ym2* | F | TTGGAGGATGGAAGTTTGGACCT |
|  | R | TGACGGTTCTGAGGAGTAGAGACCA |
| *TLR4* | F | TTCAGAGCCGTTGGTGTATC |
|  | R | CTCCCATTCCAGGTAGGTGT |
| *RAB11A* | F | ACGTCATCTCAGGGCAGTTC |
|  | R | TTGGCTTGTTCTCAGTGGTG |
| *RAB11B* | F | AGAAGCTAAAAGCCCCTTGC |
|  | R | CAACTGGCCAGCGCGGAAAG |
| *GAPDH* | F | TCAATGAAGGGGTCGTTGAT |
|  | R | CGTCCCGTAGACAAAATGGT |
| mmu-miR181a-5p | F | GGGAACATTCAACGCTGTCG |
|  | R | GTGCGTGTCGTGGAGTCG |
| mmu-miR181a-5p inhibitor | stem-loop structure | AGGUCACAAUCAACAUUCAUUGCUGUCGGUGGGUUGAACUGUGUAGAAAAGCUCACUGAACAAUGAAUGCAACUGUGGCC |
| TLR4-siRNA | F | GGACAGCUUAUAACCUUAATT |
|  | R | UUAAGGUUAUAAGCUGUCCTT |
| Rab11a-siRNA | F | GUACAGGGCUAUAACGUCUTT |
|  | R | AGACGUUAUAGCCCUGUACTT |
| Rab11b-siRNA | F | GCAUUCAGGUGGACGGCAATT |
|  |  | UUGCCGUCCACCUGAAUGCTT |
| Rictor-siRNA | F | CAGCAAACUUGUAAAGAAUTT |
|  | R | AUUCUUUACAAGUUUGCUGTT |
| TRPV2-siRNA | F | GGAUAUCUGCAACCAUCAATT |
|  | R | UUGAUGGUUGCAGAUAUCCTT |
| siRNA NC  (si-NC) | F | UUCUCCGAACGUGUCACGUTT |
|  | R | ACGUGACACGUUCGGAGAATT |

**Table S2 Gene symbols of the Top Six Enriched Exocytosis-Related GO Terms.**

| GO_P Term ID | GO_P Term Desc | Gene Symbols |
| --- | --- | --- |
| GO:0090522 | Vesicle tethering involved in exocytosis | Exoc6,Exoc4,Exoc6b |
| GO:1990182 | Exosomal secretion | Rab11a, Rab27a,Cops5,Steap3 |
| GO:1903543 | Positive regulation of exosomal secretion | Hgs,Rab7,Vps4b,Sdc1,Sdc4,Snf8,Sdcbp,Chmp2a |
| GO:0045921 | Positive regulation of exocytosis | Rab11a,Rab27a,Ncs1,Septin5,Stx1a,Stxbp1,Unc13b,Pla2g6,Atp6ap1,Rab9,Exoc2,Clasp2,Clasp1,Stxbp5,Glrx |
| GO:0043547 | Positive regulation of GTPase activity | Dock9,Lars,Rapgef1,Tbcd,Abr,Arhgap5, Rab11a,Arhgdib,Chm,Chml,Ccr7,Asap1,Dvl2,Evi5,Srgap2,Gnas,Rack1,Icam1,Itga6,Itgb1,Afdn,Mmut,Myo9b,Nf1,Bcas3,Arhgdia,Rapgef6,Ranbp2,Rasa3,Rasal1,Ralbp1,Rsu1,S100a10,Ccl2,Ccl20,Ccl22,Ccl25,Ccl3,Ccl4,Ccl5,Ccl9,Sh3bp1,Dock10,Tbc1d4,Arhgef11,Plekhg6,Flcn,Acap1,Snx13,Rin3,Rasa1,Tsc2,Vav1,Ranbp3l,Tbc1d24,Fam13b,Ric1,Rab3gap1,Rasal2,Rabgap1,Ralgapb,Arhgap24,Arhgap35,Arhgap33,Als2cl,Dock4,Pin1,Syngap1,Ralgapa2,Rgp1,Elmod2,Sipa1l2,Tbc1d8b,Srgap3,Myo9a,Prex1,Depdc5,Rabgap1l,Rasal3,Dennd1b,Agap1,Tbc1d2,Arhgef7,Rabep1,Kalrn,Net1,Gsk3b,Ralgapa1,Tbc1d1,Arfgap3,Snx9,Gapvd1,Tbc1d7,Rgs10,Dock5,Arhgef12,Arap1,Arhgap21,Als2,Rin2,Rab11fip2,Arhgap12,Dock11,Dock8,Rapgef2,Arhgap10,Acap2,Rab3gap2 |
| GO:0070588 | Calcium ion transmembrane transport | Ano6,Anxa5,Anxa6,Cacna1d,Cacna2d1,Cacnb3,Anxa2,Edn1,Ncs1,Gas6,Itgav,Itpr1,Itpr2,Itpr3,Tmem37,Pkd1,Pkd2,Psen1,Ryr1,Mcu,Tmem165,Trpv2,Tpcn2,Atp2c1,Tpcn1,Orai3,Trpm2,Cachd1,Atp2b4,Atp2a3,Sec61a1,Cacna1f,Trpm7,Asph,Atp2b1,Mcoln2,Tmco1,Anxa9,Cacng8,Mcoln1 |

**Figure S1 Preparation and characterization of nanofiber-mimetic nanorods (NMNs)**. (a) Schematic illustration of NMN synthesis via sequential in situ reduction and surface functionalization, including transformation from FeCl_3_ to FeOOH nanorods (NRs), silica coating (FeOOH@SiO_2_), formation of silica-coated superparamagnetic iron oxide nanorods (SPIONR@SiO_2_), and subsequent amine functionalization (SPIONR-NH₂). (b) Representative TEM images of NMNs obtained at each synthesis stage. (c) Vibrating sample magnetometry (VSM) analysis showing hysteresis loops of NMN after normalization to the maximum magnetic moment. (d) Zeta potential measurements of nanorods corresponding to the stages shown in (b) (n = 3 independent samples). SPIONRs that were coupled with cRGD formed NMNs.

**Figure S2 Structural characterization of SPIONRs by X-ray diffraction.** X-ray diffraction (XRD) patterns of FeOOH@SiO_2_ and SPIONR@SiO_2_. The SPIONR@SiO_2_ sample exhibits characteristic diffraction peaks consistent with the crystalline phase of Fe_3_O_4_, confirming successful conversion to SPIONRs.

**Figure S3 Schematic illustration of nanoscale isotropic matrix (NIM) construction.** (a) Maleimide-functionalized SPIONRs were conjugated to thiolated hyaluronic acid (HA) hydrogels via thiol–ene chemistry to generate SPIONR-embedded hydrogels (SPIONR-gel). (b) Cyclic RGD peptides (cRGD) were subsequently conjugated to the SPIONR-coupled matrix by consuming remaining maleimide groups, yielding ligand-functionalized NIM. Application of a lateral magnetic field induces nanorod alignment, converting NIM into a nanoscale aligned matrix (NAM).

**Figure S4 Chemical characterization of cRGD functionalization on SPIONRs.** Fourier-transform infrared (FTIR) spectra of SPIONR-NH_2_ and cRGD-functionalized SPIONRs (SPIONR-cRGD). Characteristic Fe–O stretching vibration appear at 580 cm⁻^1^. Peaks at 880 and 1090 cm⁻^1^ correspond to Si–O and Si–O–Si stretching, respectively, while the band near 1600 cm⁻^1^ is attributed to N–H scissoring. SPIONR-cRGD exhibits a pronounced C=O stretching peak at 1710 cm⁻^1^ and a shoulder at 1651 cm⁻^1^, along with amide III bands at 1380 and 1425 cm⁻^1^, confirming successful peptide conjugation.

**Figure S5 Swelling and pore characteristics of NIM and NAM hydrogels.** (a) Equilibrium swelling ratios of NIM and NAM hydrogels after incubation in PBS at 37 °C for 48 h. (b) Mean pore sizes of NIM and NAM hydrogels quantified from representative scanning electron microscopy (SEM) images. Data are presented as mean ± SD. Each point in (a) represents one independently prepared hydrogel (n = 4), whereas Each point in (b) represents the mean pore size of one independently prepared hydrogel (n = 4 hydrogels per group). At least 30 pores from five randomly selected fields were averaged for each hydrogel. Statistical significance was determined using an unpaired two-tailed Student’s *t*-test; no significant differences were detected between NIM and NAM.

**Figure S6 Analysis of ligand density stability in NIM and NAM.** (a) Confocal images of NIM and NAM that were stained by β1 integrin construct C-terminally tagged with green fluorescent protein (GFP). Scale bars are 100 µm. (b) Line profile plot of the relative fluorescent intensity of the NIM and NAM, respectively, against distance. (c) Average relative fluorescence intensity quantification. Five fields from each of three independently prepared hydrogels were analyzed, and the mean from each hydrogel was treated as one biological replicate. Data are presented as mean ± SD (n = 3). ns denotes no significant difference determined by two-tailed Student’s (t)-test.

**Figure S7. Nanoindentation-based mapping of local Young’s modulus in NIM and NAM hydrogels.** (a) Representative three-dimensional maps showing the spatial distribution of local Young’s modulus across the surfaces of the nanoscale isotropic matrix (NIM) and nanoscale aligned matrix (NAM). (b) Quantification of the mean local Young’s modulus showed no significant difference between NIM and NAM. Each dot represents an average value of one independently analyzed region. Data are presented as mean ± SD (n = 6 per group). Statistical significance was determined using an unpaired t-test. ns, not significant.

**Figure S8 Flow-cytometric gating strategy for isolation and phenotypic characterization of tumor-associated macrophages.** Tumor-derived single-cell suspensions were sequentially gated to identify cells, singlets, viable 7-AAD⁻ cells, and CD45⁺ immune cells. CD11b⁺ myeloid cells were then selected, followed by exclusion of Ly6G⁺ neutrophils. Tumor-associated macrophages were identified as CD45⁺CD11b⁺Ly6G⁻F4/80⁺ cells, while the F4/80-low population was classified as monocytes. CD86 and CD206 expression was subsequently analyzed within the macrophage gate to characterize inflammatory-like, M2-like, dual-positive, and double-negative subsets (not for isolation).

**Figure S9 Macrophage morphology analysis on NIM and NAM.** Quantification of (a) cell spreading area and (b) shape factor of macrophages on NIM or NAM after 24 h of culture. Each data point represents a single cell (20 cells from three independent hydrogels). Data are presented as mean ± SD (n = 3). Statistical significance was determined by one-way ANOVA with Tukey’s post hoc test (***p ≤ 0.001).

**Figure S10 In vivo macrophage adhesion, spreading, and polarization on implanted hydrogels.** (a) Representative fluorescence images showing macrophage adhesion and spreading on subcutaneously implanted hydrogels without polarization induction medium after 24 h. (b) Quantification of adhered cell density and cell spreading area. (c) Immunofluorescence images of iNOS and Arg-1 staining, with (d) corresponding quantitative analysis. Data are presented as mean ± SD (n = 6 mice). Adhered-cell density and spreading area were quantified from six independently implanted hydrogels in six mice. Each point represents one cell for cell spreading area or relative fluorescent intensity of iNOS/Arg-1 quantification. Statistical significance was determined by unpaired t-test (***p ≤ 0.001).

**Figure S11** **Gene expression profiling of macrophages on NIM and NAM in vivo.** Relative mRNA expression levels of iNOS, CD80, Arg-1, and Ym2 in macrophages adherent to NIM or NAM hydrogels after 24 h of subcutaneous implantation (n = 4 mice). Data are presented as mean ± SD. Statistical analysis was performed using two-way ANOVA with Sidak’s multiple-comparison test (***p ≤ 0.001).

**Figure S12 Magnetic nanoswitching dynamically regulates macrophage polarization.** Macrophages were cultured on NIM or NAM for 12 h and subsequently switched to the opposite matrix state for an additional 24 h. Confocal immunofluorescence images of iNOS (M1 marker) and Arg-1 (M2 marker), along with corresponding gene expression and cytokine secretion levels (TNF-α and IL-10), under (a) M1- or (b) M2-polarizing conditions. Scale bar: 25 μm. Data are presented as mean ± SD (n = 4). Statistical significance was determined by one-way ANOVA with Tukey’s test (**p ≤ 0.01, ***p ≤ 0.001).

**Figure S13** **Size-exclusion chromatography (SEC) purification preserves the size distribution and relative yield of TAM-derived sEVs.** Representative nanoparticle tracking analysis (NTA) profiles of sEV preparations (a) without (w/o SEC) and (b) with SEC (w SEC) purification. (c) Quantification of sEV particle concentration with SEC purification, normalized to the corresponding sample without SEC purification. Data are presented as mean ± SD from three independently prepared sEV samples. Statistical significance was determined using an unpaired two-tailed Student’s t-test. ns, not significant.

**Figure S14** **Expression of mechanosensitive TRPV channels in TAMs**. Relative mRNA expression levels of TRPV1, TRPV2, TRPV4, and TRPV6 in macrophages cultured on NIM or NAM. TRPV2 exhibited the highest expression and strongest upregulation under NAM conditions (n = 4). Data are presented as mean ± SD. Statistical significance was assessed using an unpaired t-test (**p ≤ 0.01, ***p ≤ 0.001).

**Figure S15. siRNA-mediated TRPV2 knockdown attenuates NAM-induced Ca^2+^ signaling and sEV release.** (a) Representative Western blot showing TRPV2 protein expression in NAM-primed TAMs transfected with non-targeting control siRNA (siNC) or Trpv2-targeting siRNA. β-Tubulin was used as the loading control. (b) Representative Fluo-4 AM fluorescence images and corresponding quantification of intracellular Ca^2+^ levels following TRPV2 knockdown (n = 3, two random image areas were acquired from each biological replicate). Scale bar: 25 μm. (c) Representative NTA profiles of sEVs collected from NAM-primed TAMs transfected with siNC or TRPV2-targeting siRNA. (d) Quantification of relative sEV release (n = 3). Particle concentrations were first normalized to the number of viable adherent donor cells and then expressed relative to the NAM + siNC group, which was set to 1. Data are presented as mean ± SD. Statistical significance was determined using an unpaired two-tailed Student’s *t*-test. ***p ≤ 0.001 and ****p ≤ 0.0001.

**Figure S16 Integrin β1 blockade attenuates matrix nanoalignment-induced TRPV2-associated Ca^2+^ signaling and sEV release in cRGD- and collagen I-presenting matrices.** (a) Representative confocal images of Fluo-4 AM fluorescence (green) and TRPV2 immunofluorescence (red) in TAMs cultured on cRGD-presenting NIM or NAM in the presence of an integrin β1-blocking antibody or isotype IgG control. Nuclei were counterstained with DAPI (blue). (b) Quantification of Fluo-4 AM and TRPV2 fluorescence intensities under the indicated conditions. (c) NTA quantification of relative sEV release. Particle concentrations were first normalized to the number of viable adherent donor cells and then expressed relative to the NIM + isotype IgG group, which was set to 1. (d) Representative Fluo-4 AM and TRPV2 images of TAMs cultured on recombinant collagen I-presenting NIM or NAM under the indicated treatments. (e) Quantification of Fluo-4 AM and TRPV2 fluorescence intensities. (f) NTA quantification of relative sEV release from collagen I-presenting matrices. Particle concentrations were normalized to viable adherent donor-cell number and subsequently expressed relative to the Col1-NIM + isotype IgG group, which was set to 1. Scale bars: 25 μm. Data are presented as mean ± SD (n = 3). Two random image areas were acquired from each biological replicate. Statistical significance was determined by one-way ANOVA followed by Tukey’s multiple-comparison test. **p ≤ 0.01, ***p ≤ 0.001, and ****p ≤ 0.0001.

**Figure S17 Magnetic-field exposure alone does not alter TRPV2-associated Ca^2+^ signaling or sEV release.** (a) Schematic illustration of cRGD-coated, magnetically nonresponsive FeOOH@SiO_2_ nanorods (NR-NMNs), which do not undergo alignment upon magnetic-field exposure. (b) Representative confocal images of Fluo-4 AM fluorescence (green) and TRPV2 immunofluorescence (red) in TAMs cultured on NR-NMN hydrogels in the absence or presence of a magnetic field (MF). Nuclei were counterstained with DAPI (blue). (c) Quantification of Fluo-4 AM and TRPV2 fluorescence intensities under the indicated conditions. (d) NTA quantification of relative sEV release. Particle concentrations were normalized to the number of viable adherent donor cells and then expressed relative to the NR-NMN group without magnetic-field exposure, which was set to 1. Scale bar: 25 μm. Data are presented as mean ± SD (n = 3). Two random image areas were acquired from each biological replicate. Statistical significance was determined using an unpaired two-tailed Student’s t-test; no significant differences were detected.

**Figure S18. TRPV2-dependent Ca^2+^ signaling promotes CaMKII, PYK2, and PI3K activation in NAM-primed TAMs.** Representative Western blots and densitometric quantification of (a) phosphorylated CaMKII (Thr286) relative to total CaMKII, (b) phosphorylated PYK2/PTK2B (Tyr402) relative to total PYK2/PTK2B, and (c) phosphorylated PI3K p85 relative to total PI3K p85 in NAM-primed TAMs treated with vehicle, tranilast, TRPV2 siRNA, or BAPTA-AM. β-Actin was used as the loading control. Data are presented as mean ± SD (n = 3). Statistical significance was determined by one-way ANOVA followed by Tukey’s multiple-comparison test. *p < 0.05, ***p < 0.001, and ****p < 0.0001.

**Figure S19** **Rab11a and Rab11b differentially regulate sEV secretion.** (a) Representative fluorescence images of CD63 (green) localization in macrophages following Rab11a or Rab11b knockdown. Nuclei were counterstained with DAPI (blue). Scale bar, 10 μm. (b) NTA quantification of relative sEV release. Particle concentrations were normalized to the number of viable adherent donor cells and then expressed relative to the NIM+si-NC group, which was set to 1. (c) Percentage of CD63 signal localized to the perinuclear region. Rab11a knockdown markedly reduced perinuclear CD63 accumulation and sEV release in NAM, whereas Rab11b knockdown showed minimal effects. Data are presented as mean ± SD (n = 3). Statistical significance was assessed by one-way ANOVA with Tukey’s test (**p ≤ 0.01, ***p ≤ 0.001; n.s., not significant).

**Figure S20** **PI3k inhibition suppresses NAM-induced sEV secretion.** (a) Representative fluorescence images of CD63 clustering following treatment with the PI3k inhibitor, alphelisib. Scale bar, 10 μm. (b) Quantification of perinuclear CD63 localization. (c) NTA quantification of relative sEV release. Particle concentrations were normalized to the number of viable adherent donor cells and then expressed relative to the NAM+DMSO group, which was set to 1. Data are presented as mean ± SD from three independent biological experiments (n = 3). Statistical significance was determined using an unpaired t-test (**p ≤ 0.01, ***p ≤ 0.001).

**Figure S21. LC–MS/MS proteomic profiling reveals distinct protein-cargo profiles in sEV^NIM^ and sEV^NAM^.** (a) Principal component analysis of the proteomes of sEV^NIM^ and sEV^NAM^ from three independent biological replicates. (b) Volcano plot showing differentially abundant proteins in sEV^NAM^ relative to sEV^NIM^. (c) Row-wise z-score heatmap of representative pro-inflammatory proteins enriched in sEV^NIM^ and selected immunoregulatory, metabolic, and tissue-remodeling proteins enriched in sEV^NAM^. (d) GO-based gene set enrichment analysis comparing sEV^NAM^ with sEV^NIM^. Positive normalized enrichment scores indicate pathways enriched in sEV^NAM^, whereas negative scores indicate pathways enriched in sEV^NIM^. Dot size represents gene-set size, and color indicates the adjusted p-value (n = 3).

**Figure S22 Reciprocal genetic perturbation supports partially separable Rab11a and mTORC2-associated signaling outputs in NAM-primed TAMs.** (a) Representative Western blots of total mTOR, phosphorylated mTOR at Ser2481, GTP-bound Rab11a (GTP-Rab11a), and total Rab11a in NAM-primed TAMs transfected with non-targeting control siRNA (siNC) or Rab11a-targeting siRNA (Rab11a-siRNA). β-Tubulin was used as the loading control. Rab11a knockdown markedly reduced total and GTP-Rab11a but did not significantly alter mTOR Ser2481 phosphorylation. (b) Representative Western blots of the corresponding proteins in NAM-primed TAMs transfected with si-NC or Rictor-targeting siRNA (Rictor-siRNA). Rictor-targeting siRNA treatment reduced mTOR Ser2481 phosphorylation without significantly affecting GTP-Rab11a. (c) Statistical quantification of Rab11a activation, expressed as GTP-Rab11a relative to total Rab11a, and mTORC2-associated activation, expressed as phosphorylated mTOR Ser2481 relative to total mTOR. Data are presented as mean ± SD from (n = 3). Statistical significance was determined by one-way ANOVA followed by Tukey’s multiple-comparison test. ns, not significant; *p < 0.05 and **p < 0.01.

**Figure S23 Determination of cell viability by CCK-8 assays at 0, 24, 48, and 72 h after the sEV treatments.** Data are presented as mean ± SD (n = 3).

**Figure S24** **miR-181a-5p mediates sEV^NAM^-induced macrophage M2 polarization.** Quantification of CD68⁺CD206⁺ (M2-like) macrophages treated with sEV^NAM^ or miR-181a-5p–deficient sEV^NAM^ under (a) M1- or (b) M2-polarizing conditions for 36 h. sEV^NAM+miR-181a-5p KD^ was derived from donor cells with miR-181a-5p knockdown; NC denotes negative control. Cells cultured in EV-depleted FBS without sEV supplementation served as controls. Data are presented as mean ± SD from n = 3 independent biological experiments. Statistical significance was determined by one-way ANOVA with Tukey’s test (*p ≤ 0.05, **p ≤ 0.01, ***p ≤ 0.001).

**Figure S25. IL-10/TGF-β pathway blockade and miR-181a-5p depletion cooperatively attenuate sEV NAM-induced M2-like macrophage polarization with the presence of M1 stimulators.** (a) Representative immunofluorescence images of Arg-1 (red) and iNOS (magenta) in recipient macrophages treated with sEV^NAM^, sEV^NAM^ plus IL-10-neutralizing antibody (NAb), sEV^NAM^ + SB-431542, miR-181a-5p-depleted sEV^NAM^, or the corresponding combined treatments. F-actin and nuclei were counterstained with phalloidin (green) and DAPI (blue), respectively. (b) Quantification of iNOS and Arg-1 fluorescence intensities. Data are presented as mean ± SD (n = 3). Statistical significance was determined by one-way ANOVA followed by Tukey’s multiple-comparison test. *p < 0.05, **p < 0.01, ***p < 0.001, and ****p < 0.0001.

**Figure S26 In vivo effects of sEV^NIM^ and sEV^NAM^ on macrophage polarization and skin remodeling.** (a) Experimental timeline for subcutaneous sEV administration. sEVs were injected every 3 days starting on Day 1, and skin tissues were collected on Day 14; PBS injection served as a control. SubQ denotes “subcutaneous”. (b) Representative immunofluorescence images of CD68, CD86, and CD206 staining in the dermal layer. Scale bars, 20 μm and 200 μm. (c) Quantification of CD68⁺CD86⁺ (M1) and CD68⁺CD206⁺ (M2) macrophages (n = 6 mice; two random regions per mouse). Data are presented as mean ± SD. Statistical significance was determined by one-way ANOVA with Tukey’s test (*p ≤ 0.05, **p ≤ 0.01, ***p ≤ 0.001).

**Figure S27 sEV-mediated regulation of collagen deposition and dermal thickness.** (a) Representative hematoxylin and eosin (H&E; upper panels) and Masson’s trichrome (lower panels) staining of skin tissues collected on Day 14. PBS injection served as control. Scale bars, 200 μm (H&E) and 100 μm (Masson’s). (b) Quantification of collagen index and (c) average dermal thickness across treatment groups. Note that this assay was used only to evaluate the local tissue response to sEV administration and not to infer a syngeneic tumor-immune interaction. Data are presented as mean ± SD (n = 6, two random regions per mouse). Statistical significance was assessed using one-way ANOVA with Tukey’s test (*p ≤ 0.05, **p ≤ 0.01, ***p ≤ 0.001).

**Figure S28 In vivo biodistribution of intravenously administered sEVs derived from NIM- (sEV^NIM^) and NAM-primed (sEV^NAM^) TAMs.** (a) Representative longitudinal IVIS images of B16-F10 melanoma-bearing mice at 0, 2, 6, 24, and 48 h after intravenous administration of DiR-labeled sEV^NIM^ or sEV^NAM^. White arrowheads indicate the tumor region. (b) Quantification of fluorescence intensity within the predefined tumor-associated region of interest over 48 h. (c) Representative *ex vivo* fluorescence images of the heart, liver, spleen, lung, kidney, and tumor collected from mice at 24 and 48 h after sEV administration. Samples shown at 0 h represent uninjected background controls. (d) Quantification of ex vivo fluorescence intensity in the tumor, spleen, and liver. Both sEV^NIM^ and sEV^NAM^ accumulated predominantly in the liver and spleen, with detectable tumor-associated fluorescence at 24 and 48 h. Data are presented as mean ± SD (n = 3). Statistical significance was analyzed by two-way ANOVA followed by Tukey’s multiple-comparisons test. ****p ≤ 0.0001.

**Figure S29. Systemically administered sEV^NAM^ exhibits enhanced association with CD68⁺CD163⁺ TAMs in melanoma tissue.** (a) Representative confocal images of melanoma sections collected after intravenous administration of fluorescently labeled sEV^NIM^ or sEV^NAM^. Labeled sEVs are shown in green, CD68 in red, CD163 in yellow, and nuclei in blue. Merged images show the spatial association of sEV fluorescence with CD68⁺CD163⁺ TAM-rich regions. (b) Quantification of sEV colocalization with CD68⁺CD163⁺ TAMs using Manders’ overlap coefficient. Measurements from multiple fields within each tumor were averaged to generate one value per mouse. Data are presented as mean ± SD (n = 3). Statistical significance was determined using an unpaired two-tailed Student’s t-test. ****p ≤ 0.0001.

**Figure S30 KEGG pathway enrichment analysis by miR-181a-5p derived from sEV^NAM^.** Top 20 enriched KEGG pathways associated with genes upregulated by miR-181a-5p.

**Figure S31 Flow-cytometric analysis confirms preferential in vivo uptake of M2pep-modified lipid nanocarriers by CD206⁺ TAMs.** Cou-6-labeled M2pep-modified lipid nanocarriers were intravenously administered to melanoma-bearing mice, and tumors were collected 24 h later for flow-cytometric analysis. Following exclusion of debris, doublets, and dead cells, cellular subsets were identified using the gating strategy shown in Figure S8. CD206⁺ TAMs were defined as CD45⁺CD11b⁺Ly6G⁻F4/80⁺CD206⁺ cells, CD86⁺ myeloid cells as CD45⁺CD11b⁺Ly6G⁻CD86⁺ cells, and tumor-cell-enriched cells as CD45⁻FSC^high^ cells. Cou-6 positivity within each subset was determined using unlabeled and fluorescence-minus-one controls. (a) Percentage of Cou-6⁺ cells within each indicated subset. (b) Proportion of the total viable tumor-derived cell population represented by Cou-6⁺ cells from each subset. Cou-6-labeled lipid nanocarriers were detected predominantly in CD206⁺ TAMs, supporting preferential, although not exclusive, delivery to this population. Data are presented as mean ± SD (n = 3 mice). Statistical significance was determined by one-way ANOVA followed by Tukey’s multiple-comparison test. *p ≤ 0.05, **p ≤ 0.01, and ***p ≤ 0.001.

**References**

(1) Wong, S. H. D.; Yin, B.; Li, Z.; Yuan, W.; Zhang, Q.; Xie, X.; Tan, Y.; Wong, N.; Zhang, K.; Bian, L. Mechanical manipulation of cancer cell tumorigenicity via heat shock protein signaling. *Sci. Adv.* **2023**, *9* (27), eadg9593.

(2) Li, Z.; Jin, J.; Yang, F.; Song, N.; Yin, Y. Coupling magnetic and plasmonic anisotropy in hybrid nanorods for mechanochromic responses. *Nat. Commun.* **2020**, *11* (1), 2883.

(3) Wong, S. H. D.; Yin, B.; Yang, B.; Lin, S.; Li, R.; Feng, Q.; Yang, H.; Zhang, L.; Yang, Z.; Li, G. Anisotropic nanoscale presentation of cell adhesion ligand enhances the recruitment of diverse integrins in adhesion structures and mechanosensing‐dependent differentiation of stem cells. *Adv. Funct. Mater.* **2019**, *29* (8), 1806822.

(4) Lane, R. E.; Korbie, D.; Trau, M.; Hill, M. M. Optimizing size exclusion chromatography for extracellular vesicle enrichment and proteomic analysis from clinically relevant samples. *Proteomics* **2019**, *19* (8), 1800156.

(5) Korani, M.; Ghaffari, S.; Attar, H.; Mashreghi, M.; Jaafari, M. R. Preparation and characterization of nanoliposomal bortezomib formulations and evaluation of their anti-cancer efficacy in mice bearing C26 colon carcinoma and B16F0 melanoma. *Nanomedicine* **2019**, *20*, 102013.

(6) Hirai, Y.; Saeki, R.; Song, F.; Koide, H.; Fukata, N.; Tomita, K.; Maeda, N.; Oku, N.; Asai, T. Charge-reversible lipid derivative: A novel type of pH-responsive lipid for nanoparticle-mediated siRNA delivery. *Int. J. Pharm.* **2020**, *585*, 119479.
